# Supplementary figures and images for: Dynamic proteomic and phosphoproteomic atlas of corticostriatal axons in neurodevelopment
Source: eLife. 2022 Oct 14;11:e78847. doi: 10.7554/eLife.78847 (PMC9629834; doi:10.7554/eLife.78847)

Figure 1b

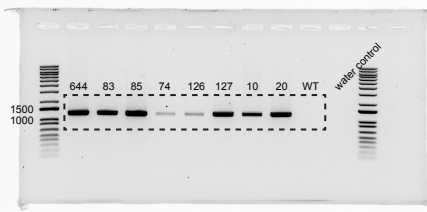

Figure 1e

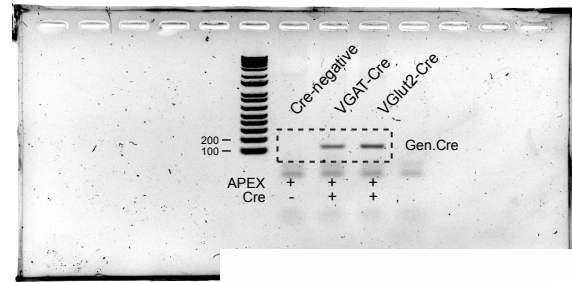

Figure 1c

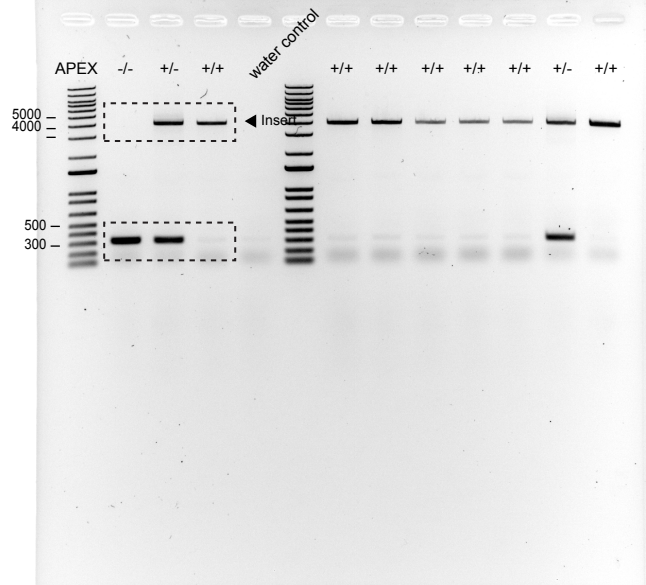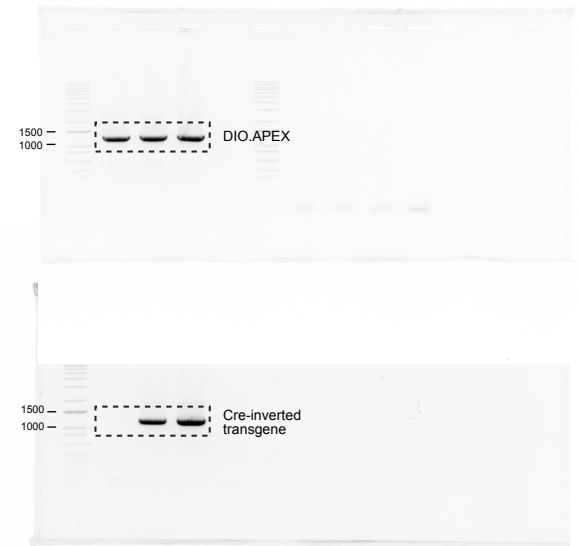

Figure 1d

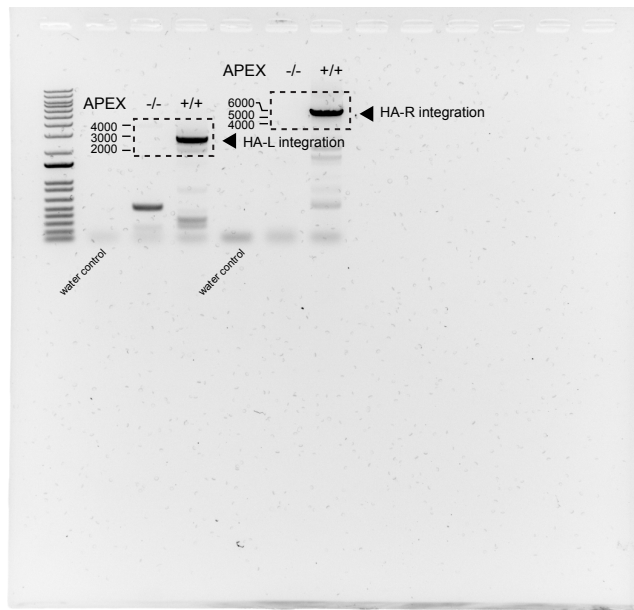

Supplement: Figure 1—source data 1. [file elife-78847-fig1-data1.zip › Figure1-source data 1/Figure1_source_data1.pdf]

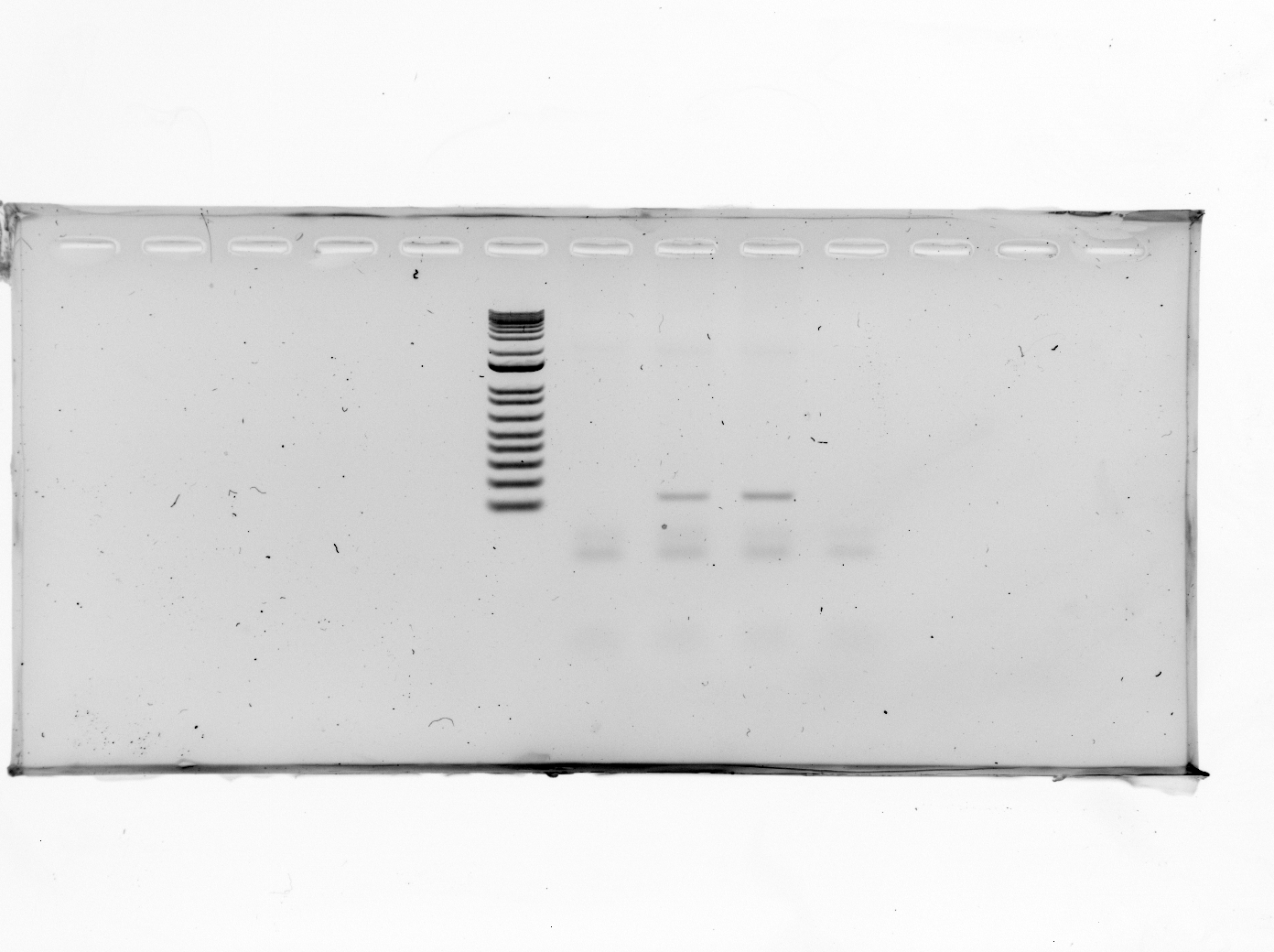

Supplement: Figure 1—source data 1. [file elife-78847-fig1-data1.zip › Figure1-source data 1/figure1_sourcedata_1_figure1e_GenCre.tif]

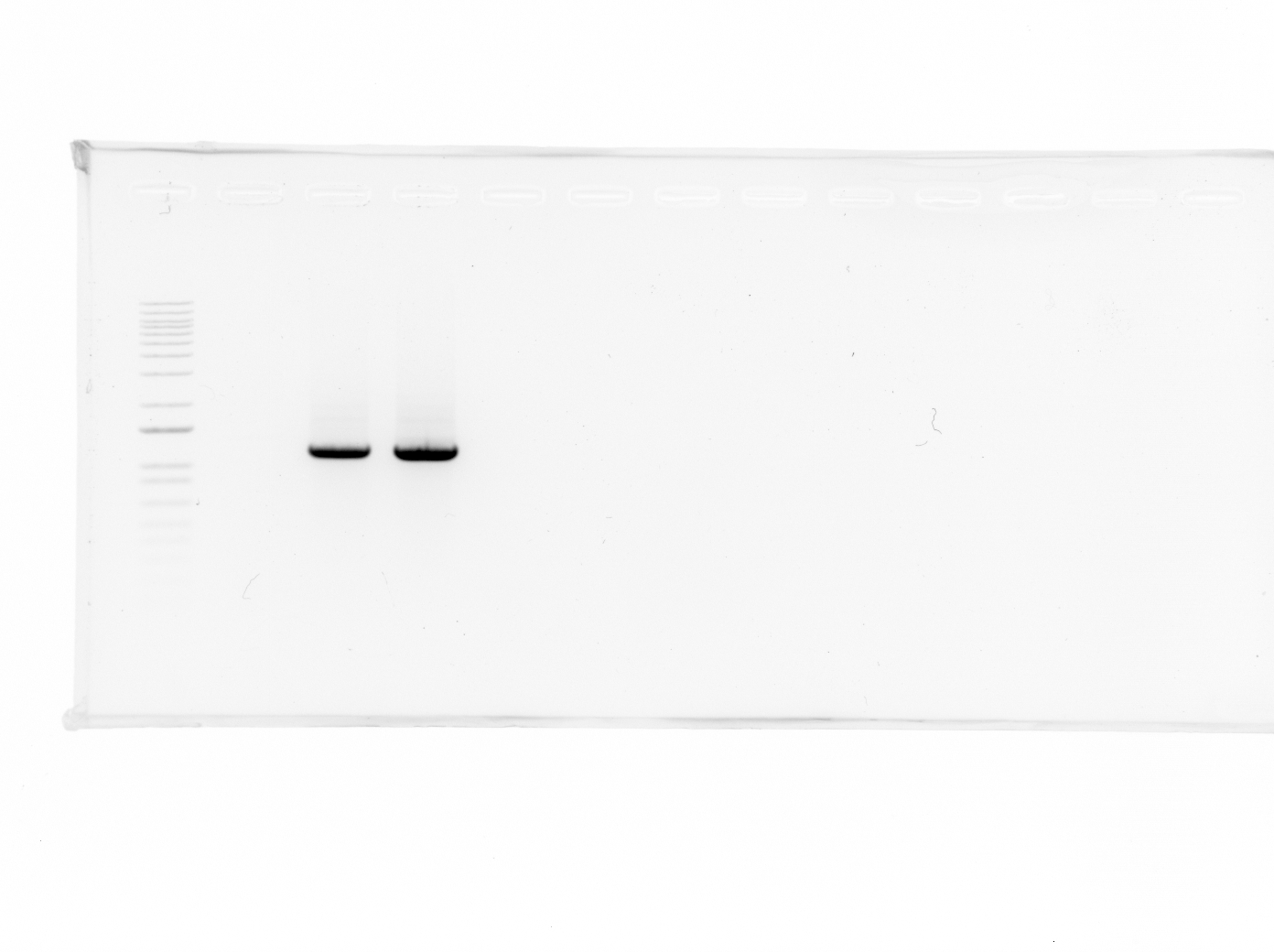

Supplement: Figure 1—source data 1. [file elife-78847-fig1-data1.zip › Figure1-source data 1/figure1_sourcedata_1_figure1e_CreInverted.tif]

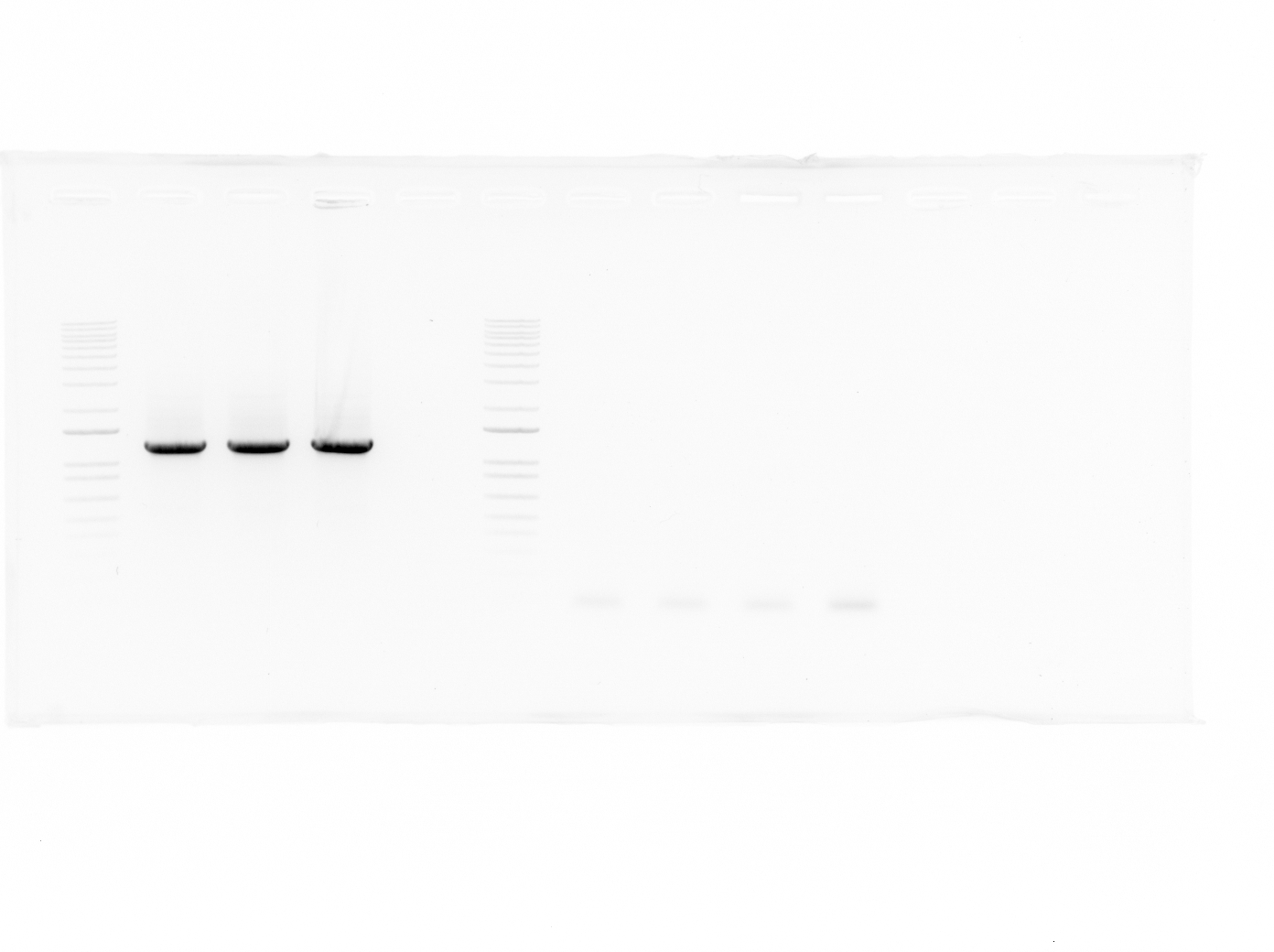

Supplement: Figure 1—source data 1. [file elife-78847-fig1-data1.zip › Figure1-source data 1/figure1_sourcedata_1_figure1e_DIOAPEX.tif]

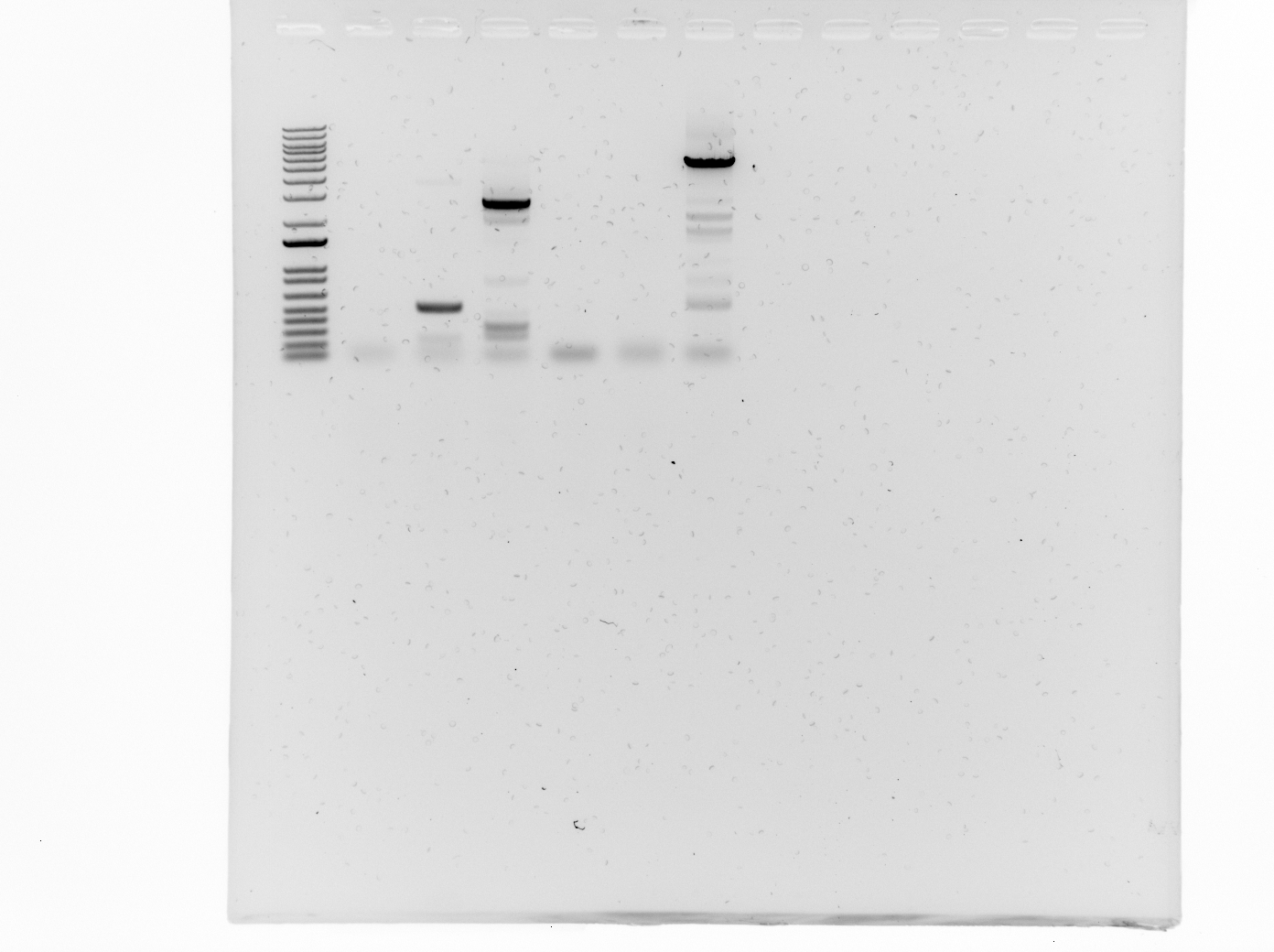

Supplement: Figure 1—source data 1. [file elife-78847-fig1-data1.zip › Figure1-source data 1/figure1_sourcedata_1_figure1d.tif]

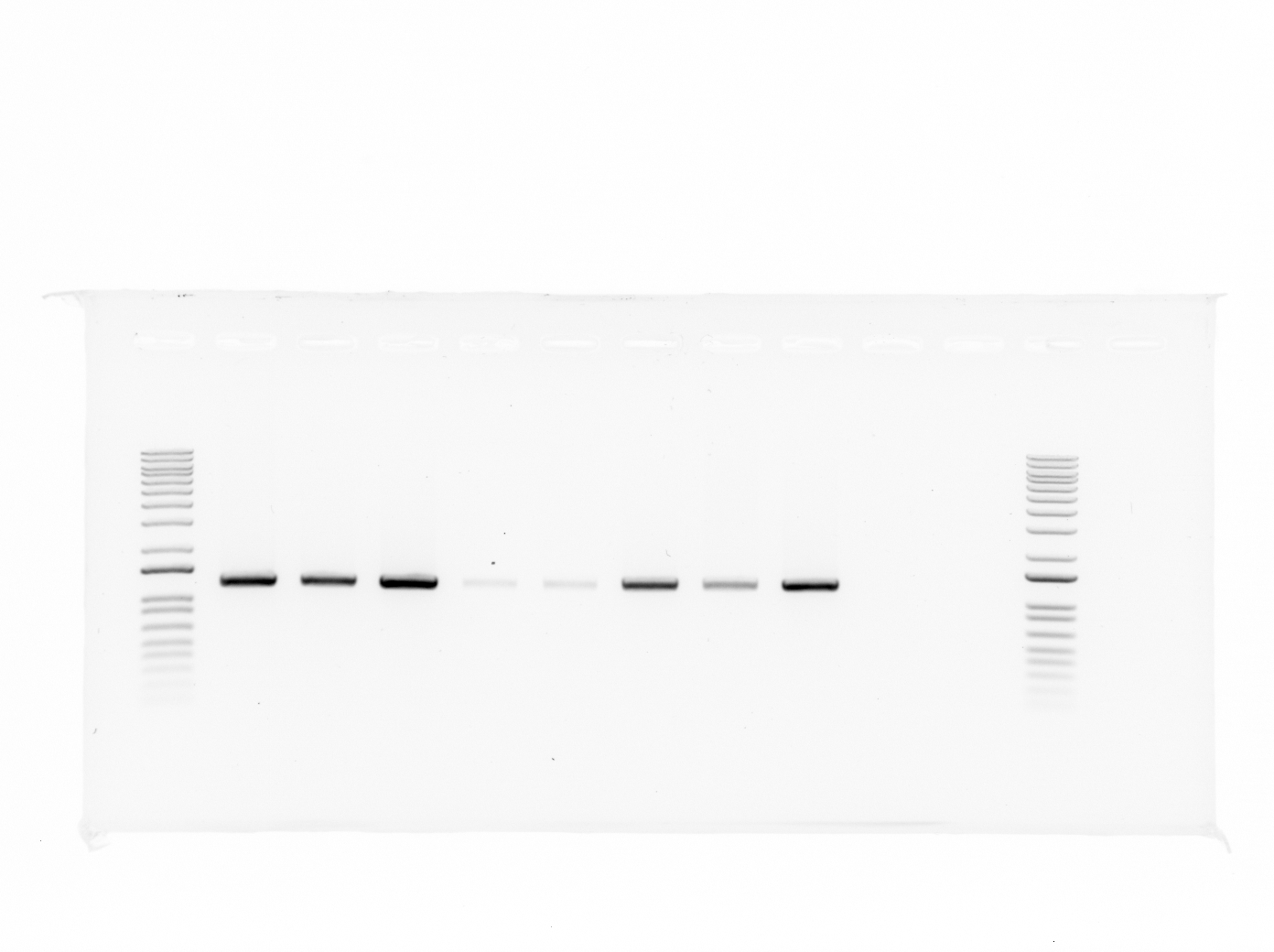

Supplement: Figure 1—source data 1. [file elife-78847-fig1-data1.zip › Figure1-source data 1/figure1_sourcedata_1_figure1b.tif]

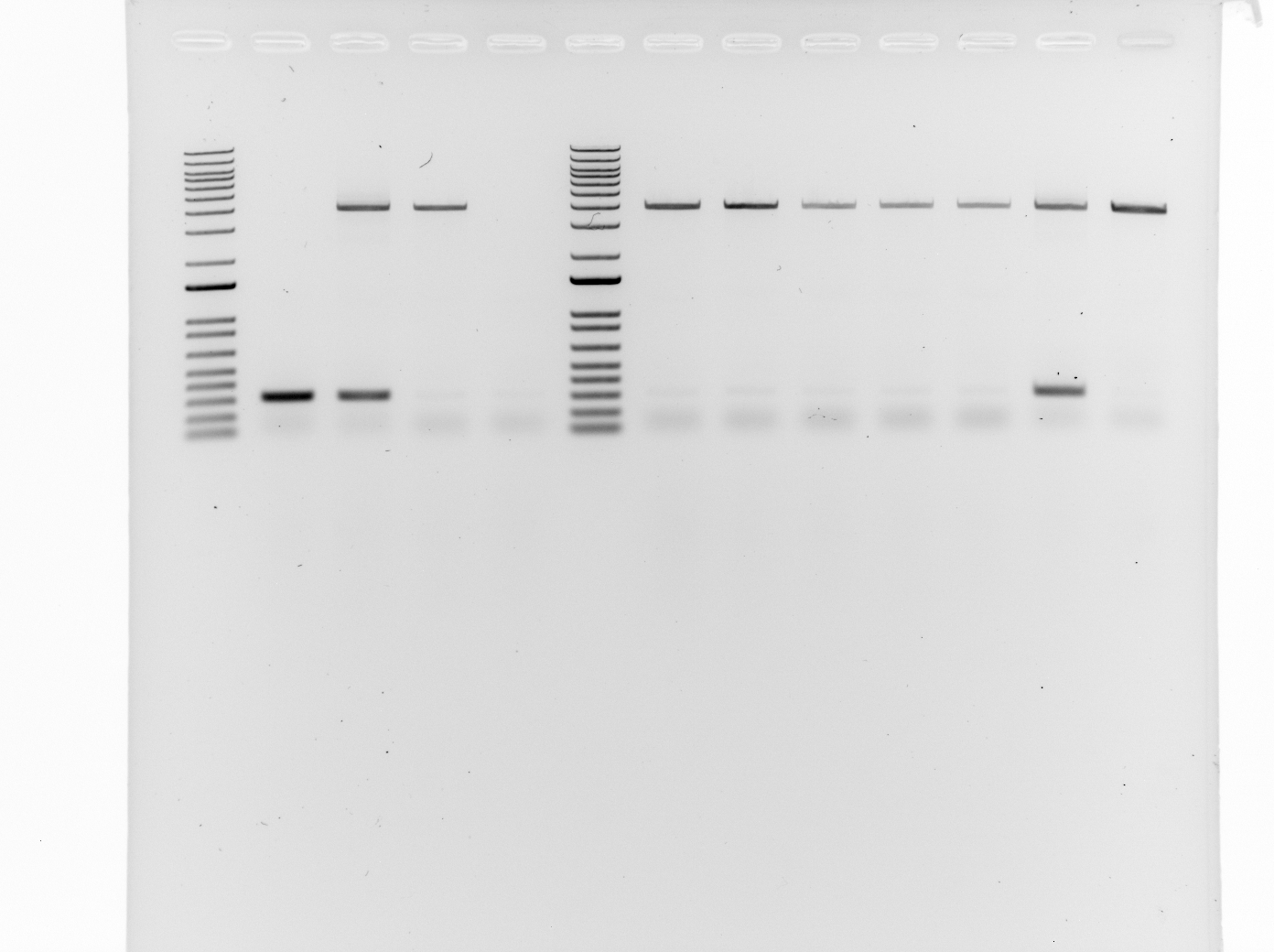

Supplement: Figure 1—source data 1. [file elife-78847-fig1-data1.zip › Figure1-source data 1/figure1_sourcedata_1_figure1c.tif]

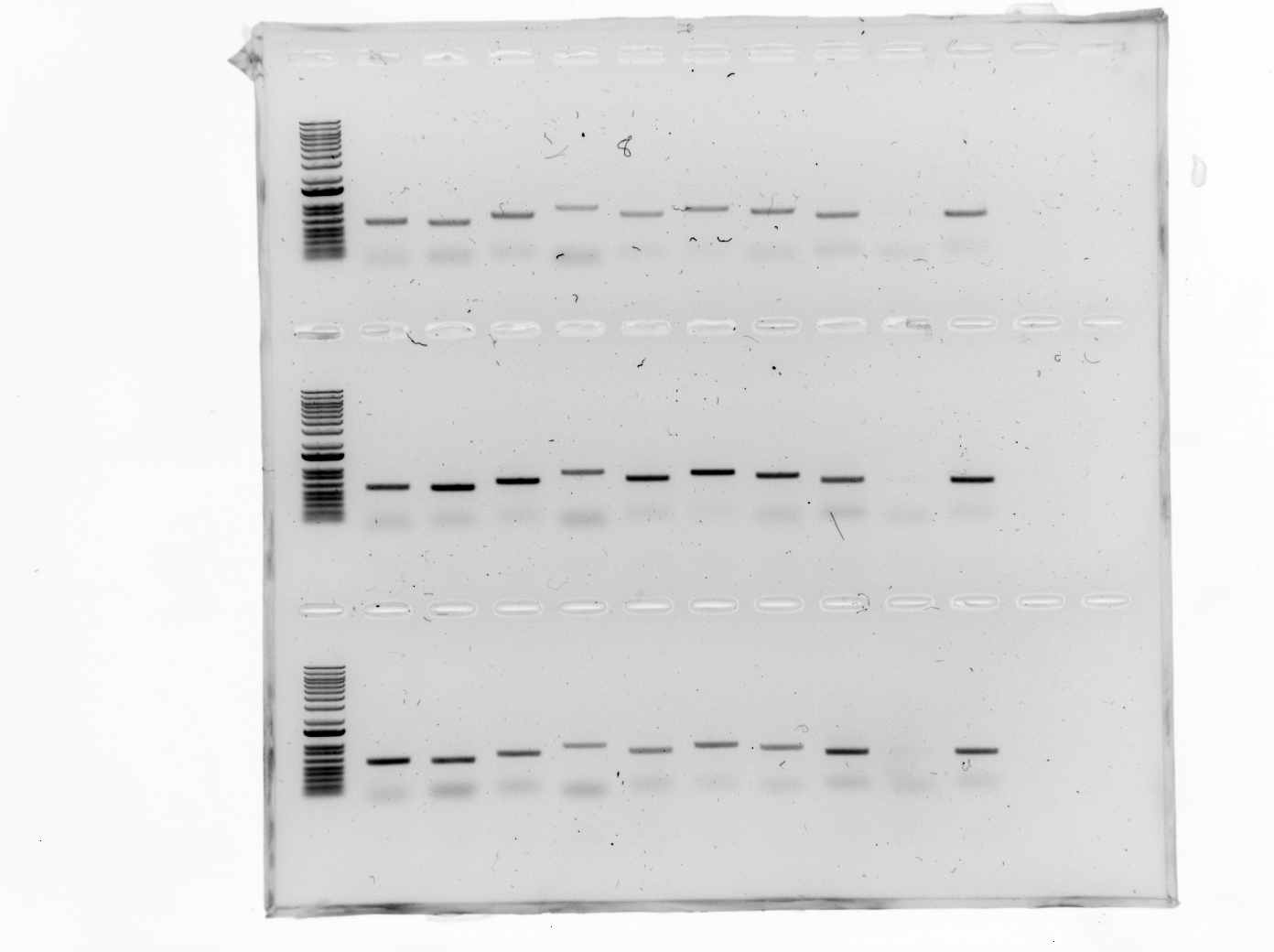

Supplement: Figure 1—figure supplement 1—source data 1. [file elife-78847-fig1-figsupp1-data1.zip › Figure1-supplement1-source data 1/Figure1_supplement1a_source_data1_offtargets1-10_mouse_83_644_85.tif]

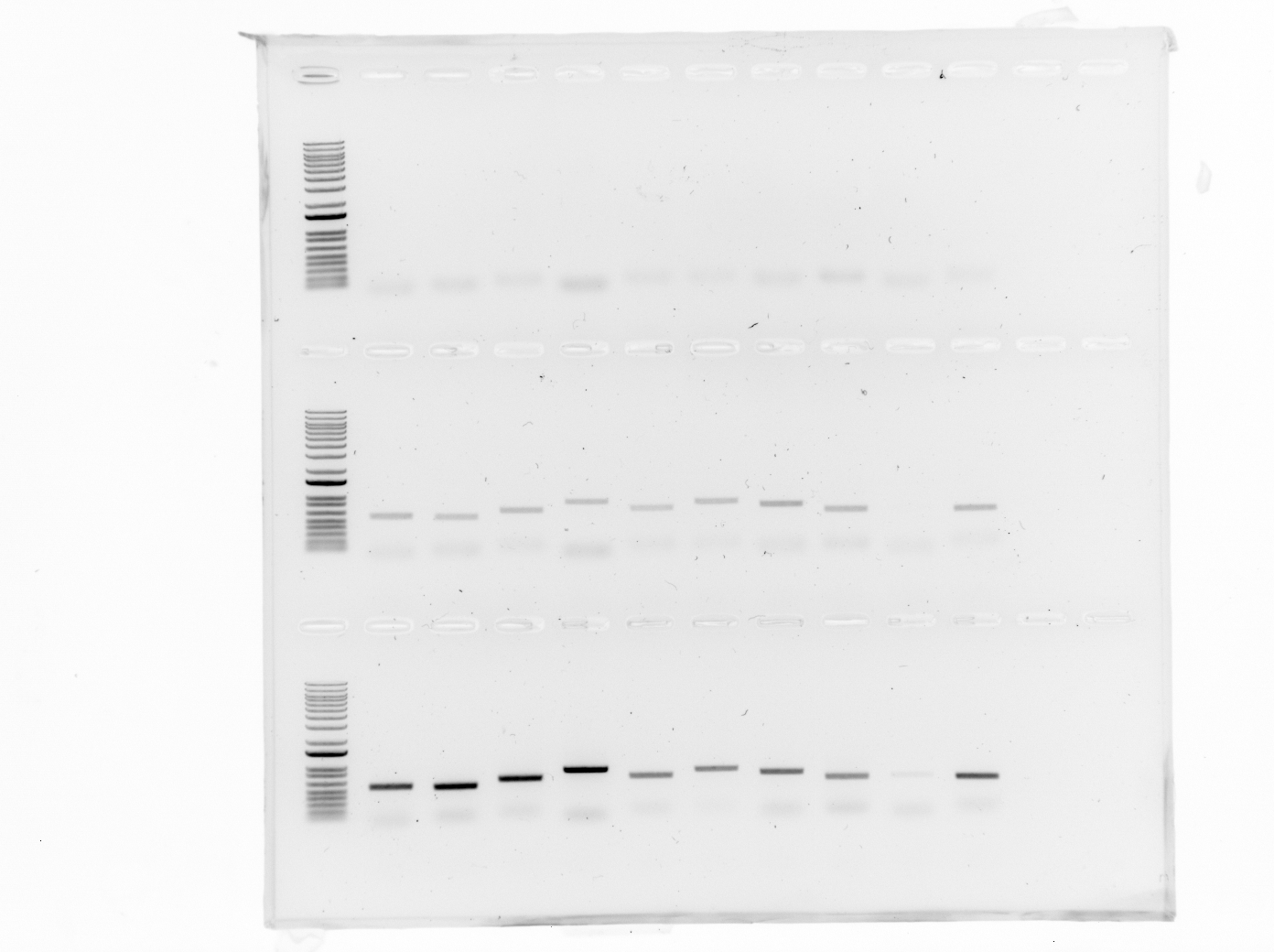

Supplement: Figure 1—figure supplement 1—source data 1. [file elife-78847-fig1-figsupp1-data1.zip › Figure1-supplement1-source data 1/Figure1_supplement1a_source_data1_offtargets1-10_mouse_74_127_WT.tif]

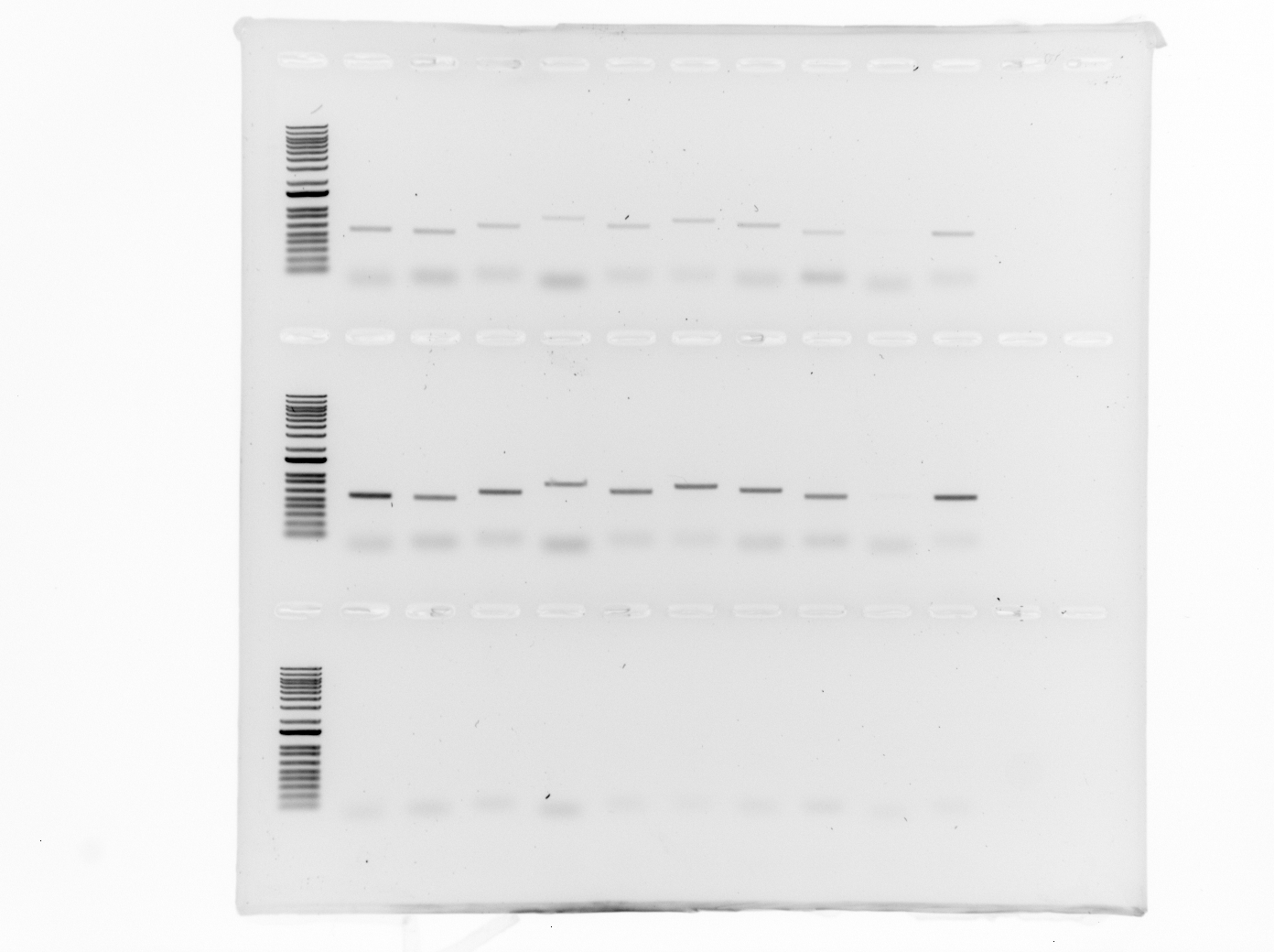

Supplement: Figure 1—figure supplement 1—source data 1. [file elife-78847-fig1-figsupp1-data1.zip › Figure1-supplement1-source data 1/Figure1_supplement1a_source_data1_offtargets1-10_mouse_10_20_126.tif]

Figure 1 - Figure supplement 2a

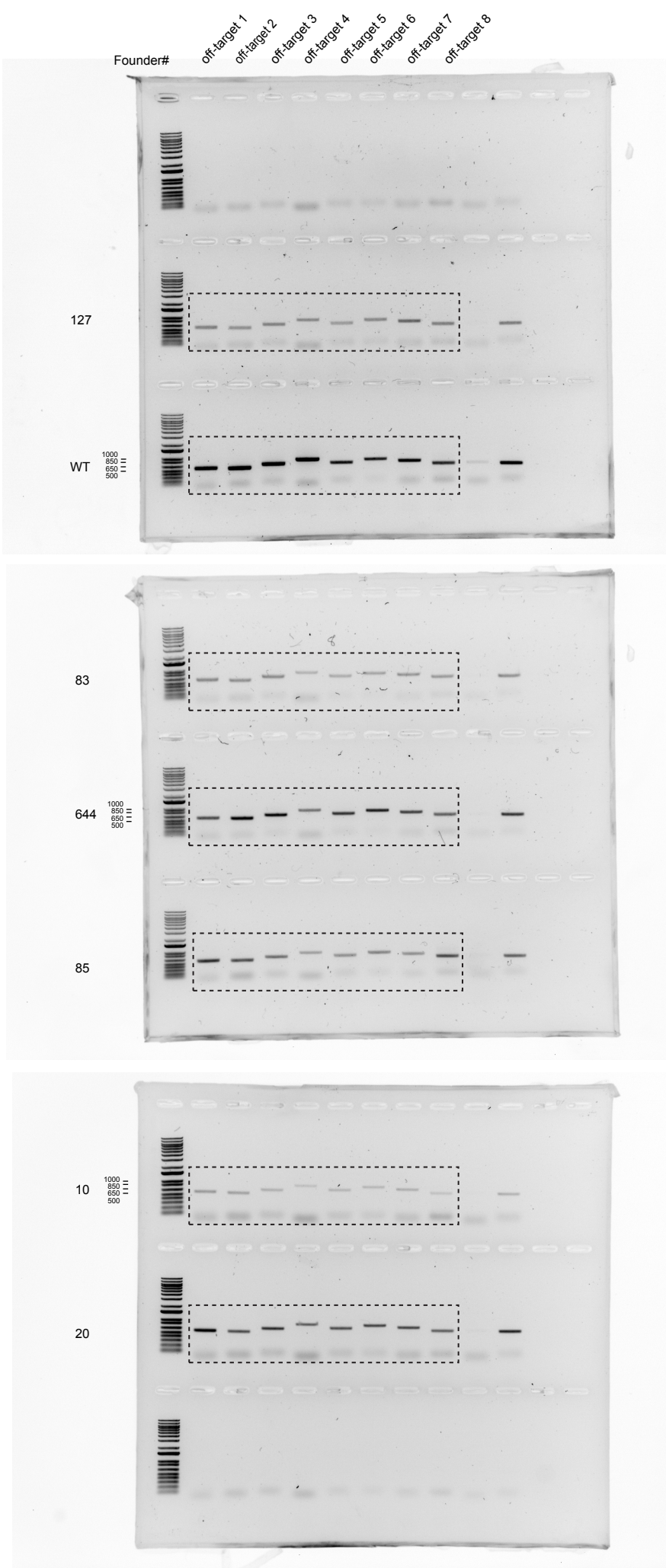

Supplement: Figure 1—figure supplement 1—source data 1. [file elife-78847-fig1-figsupp1-data1.zip › Figure1-supplement1-source data 1/Figure1_supplement1_source_data1.pdf]

Figure 2c

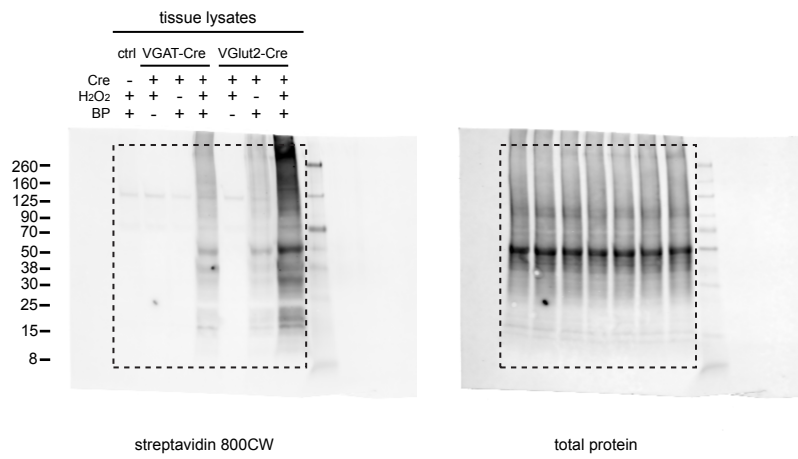

Figure 2d

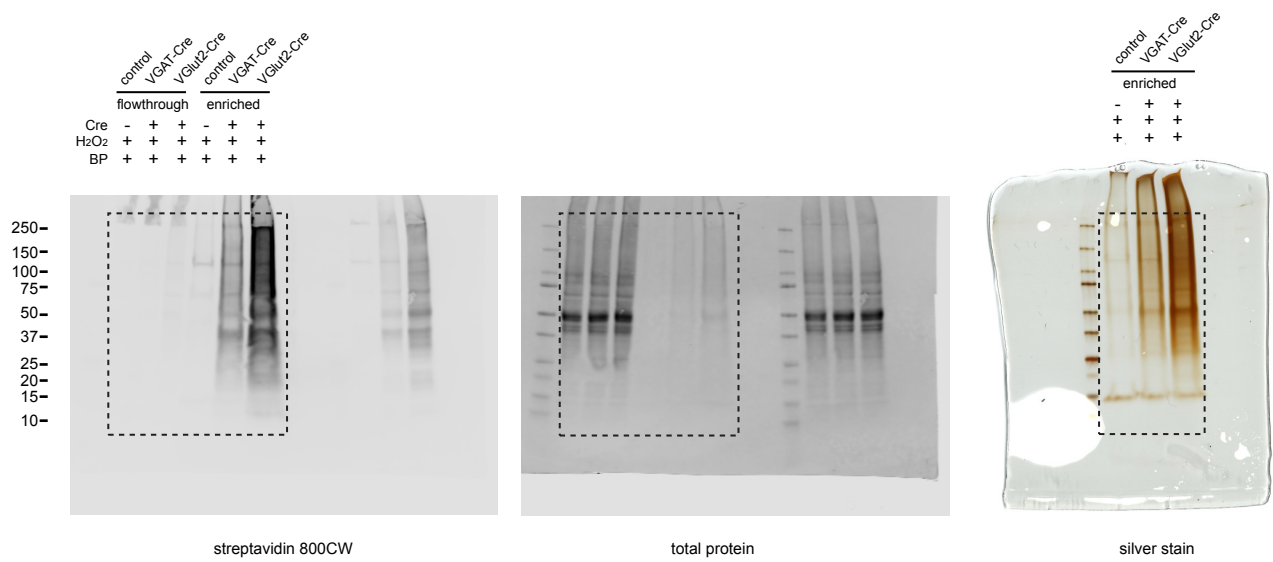

Supplement: Figure 2—source data 1. [file elife-78847-fig2-data1.zip › Figure2-source data 2/Figure2_source_data1.pdf]

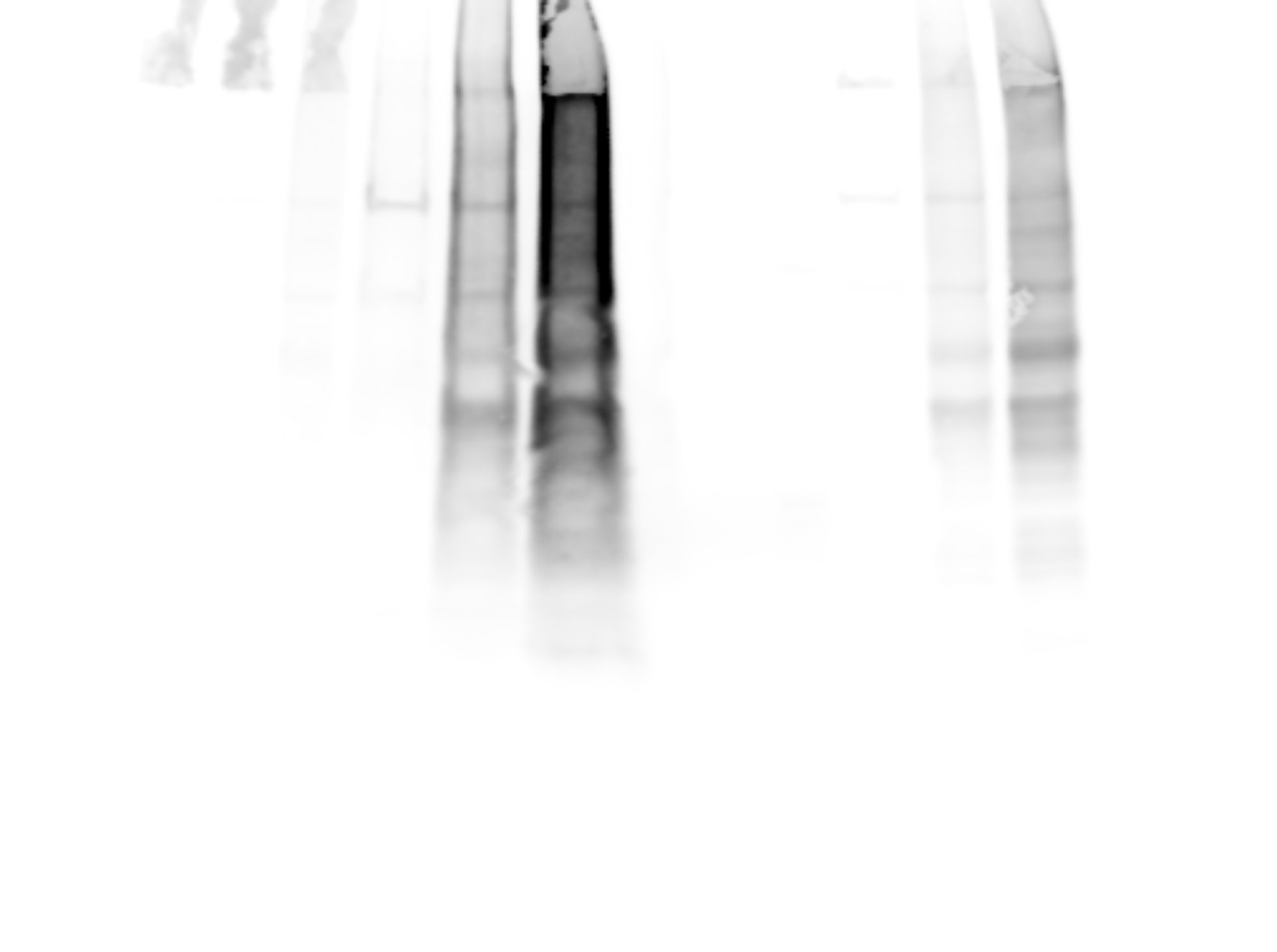

Supplement: Figure 2—source data 1. [file elife-78847-fig2-data1.zip › Figure2-source data 2/figure2_source_data_1_figure2d_streptavidin.tif]

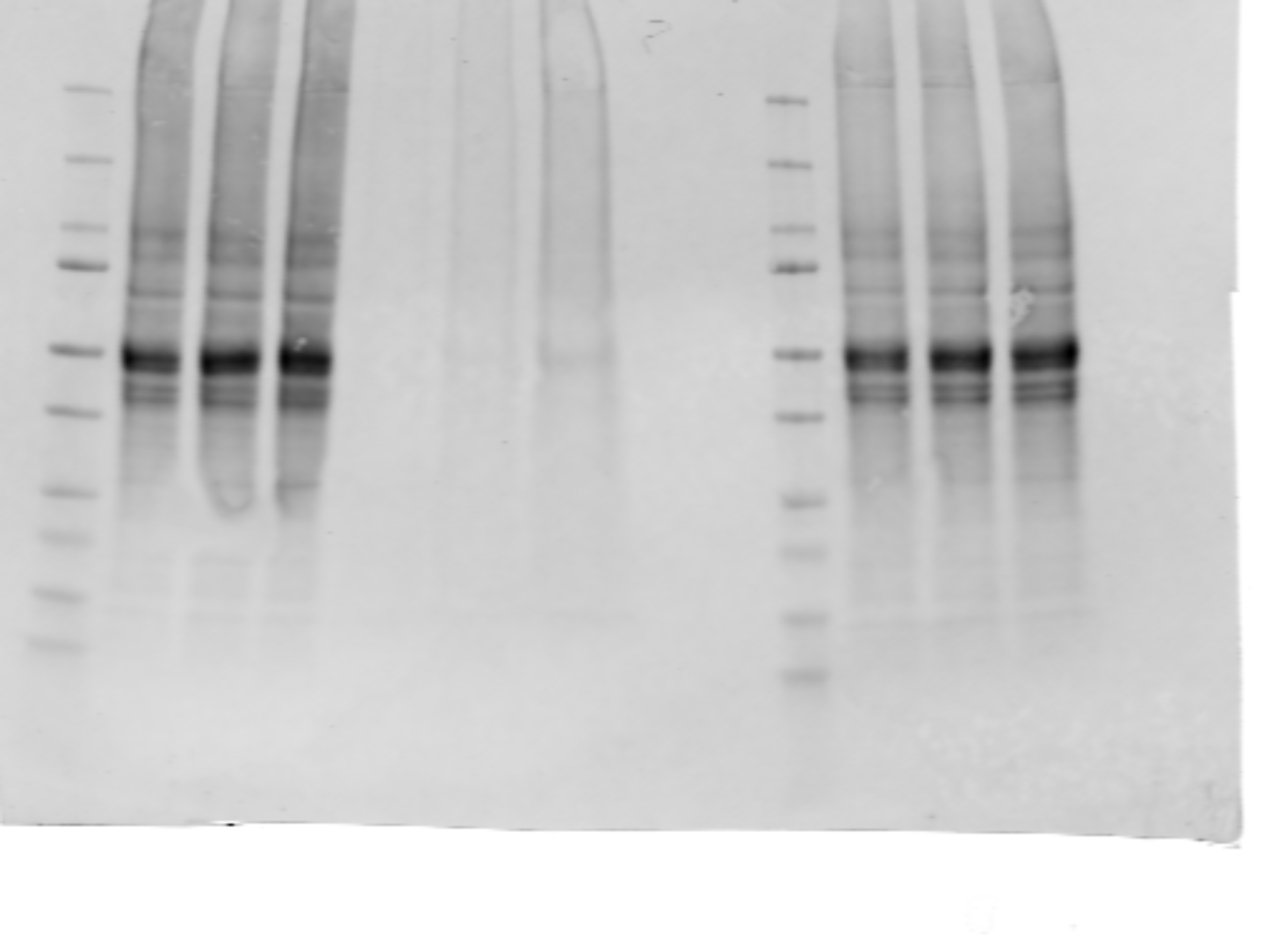

Supplement: Figure 2—source data 1. [file elife-78847-fig2-data1.zip › Figure2-source data 2/figure2_source_data_1_figure2d_REVERT.tif]

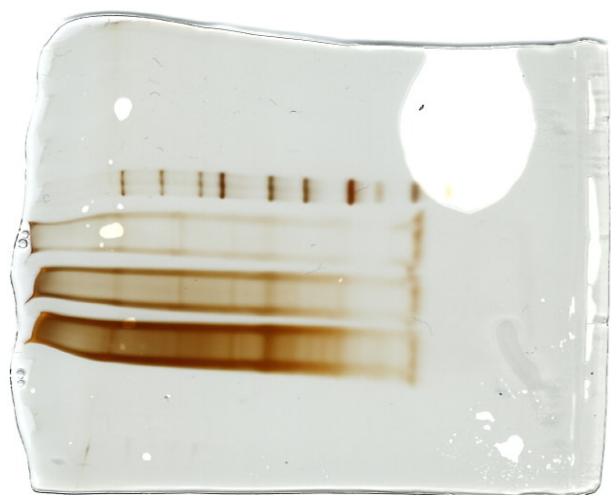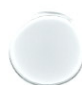

Supplement: Figure 2—source data 1. [file elife-78847-fig2-data1.zip › Figure2-source data 2/figure2_source_data_1_figure2d_silverstain.PDF]

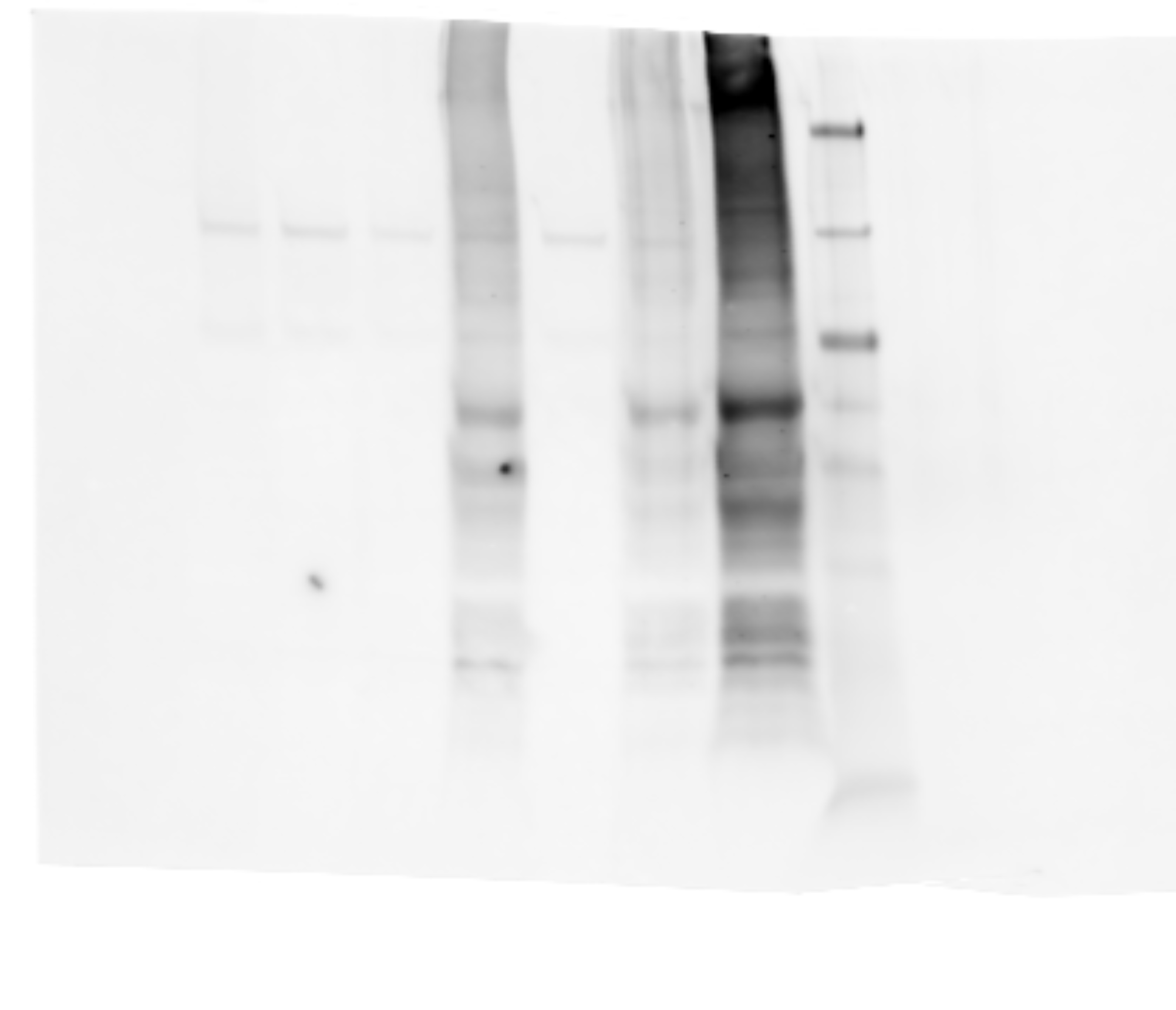

Supplement: Figure 2—source data 1. [file elife-78847-fig2-data1.zip › Figure2-source data 2/figure2_source_data_1_figure2c_streptavidin.tif]

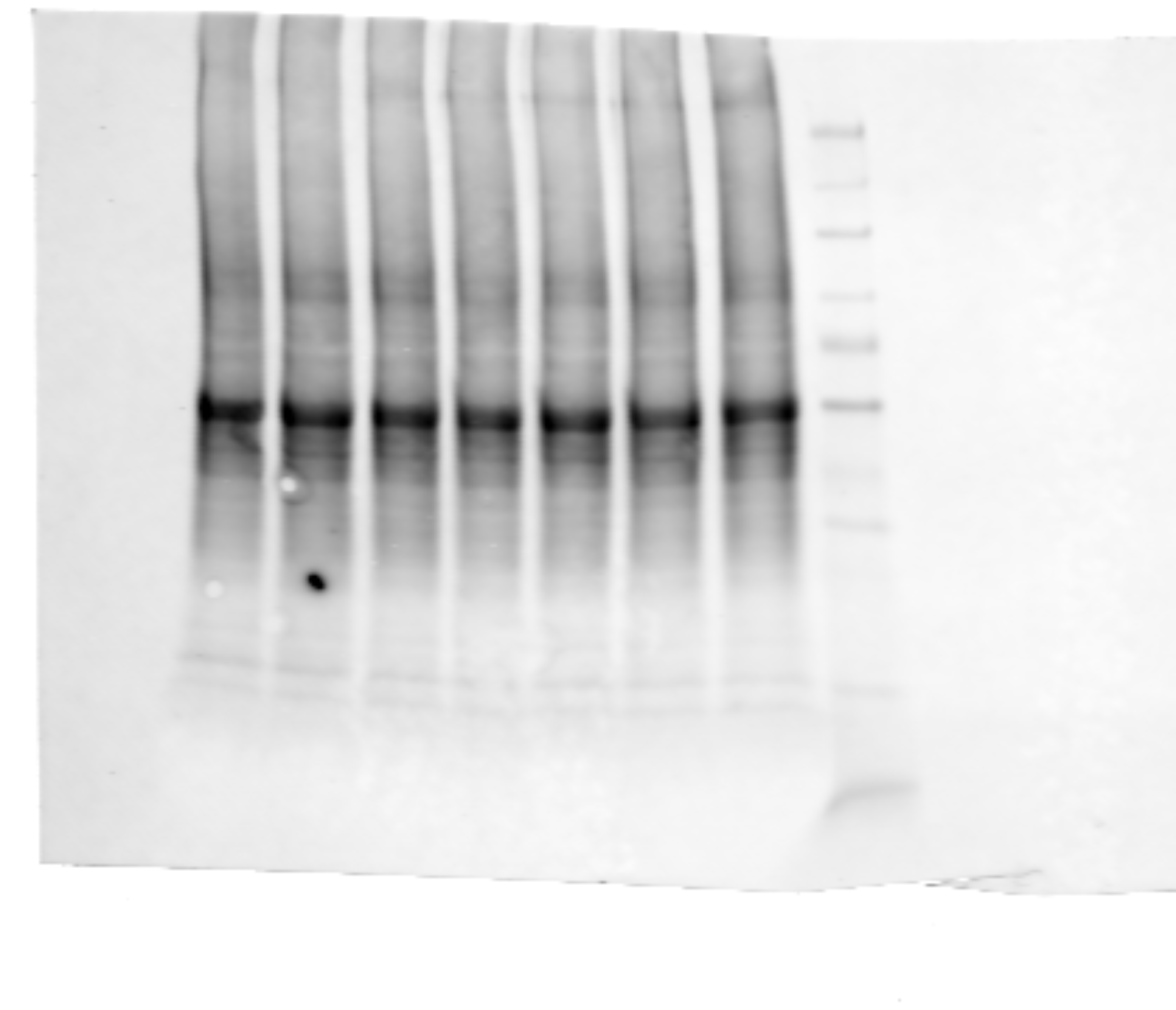

Supplement: Figure 2—source data 1. [file elife-78847-fig2-data1.zip › Figure2-source data 2/figure2_source_data_1_figure2c_REVERT.tif]

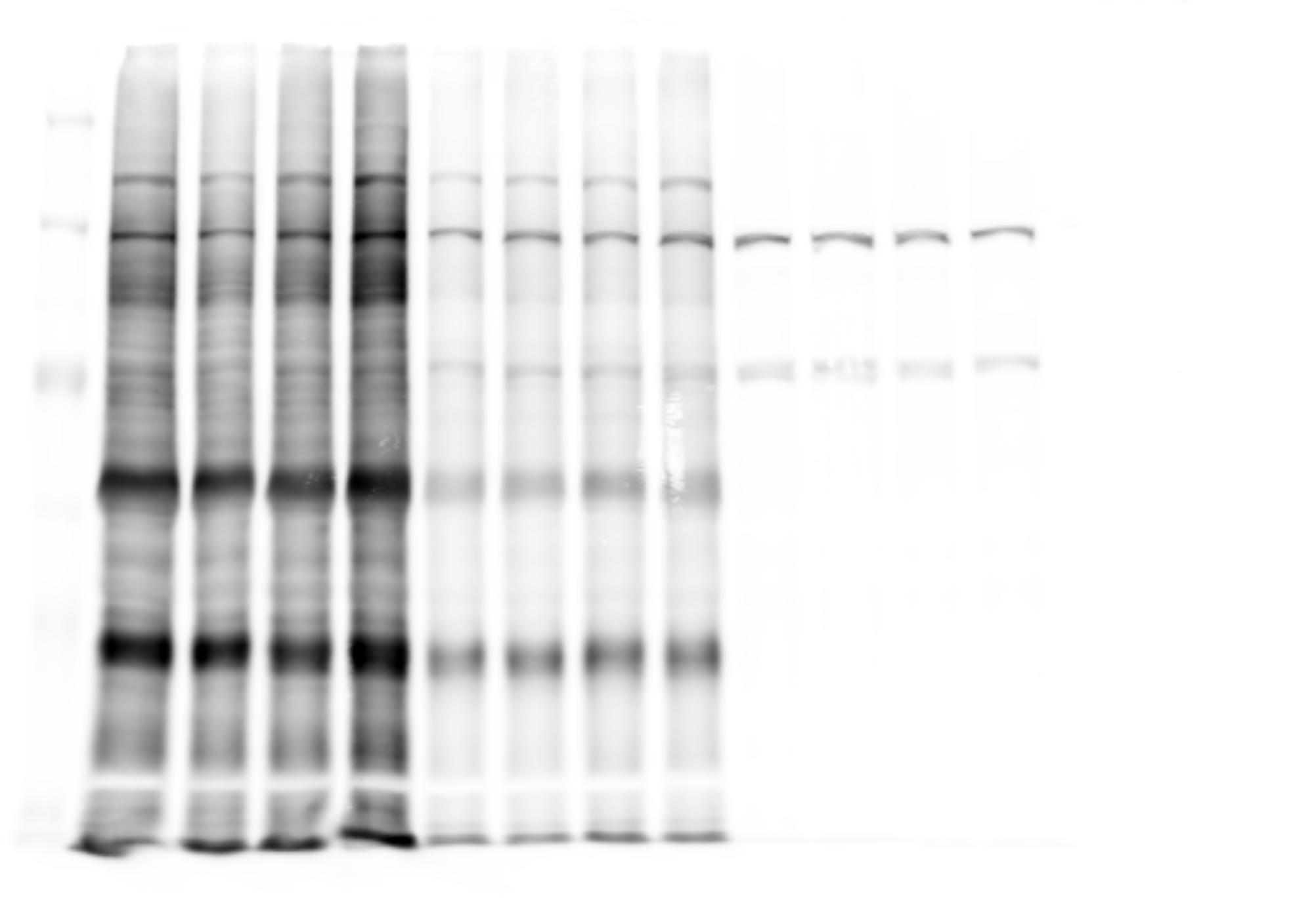

Supplement: Figure 2—figure supplement 1—source data 1. [file elife-78847-fig2-figsupp1-data1.zip › Figure2-supplement1-source data 1/Figure2_supplement1_source_data1_supplement1b_streptavidin.tif]

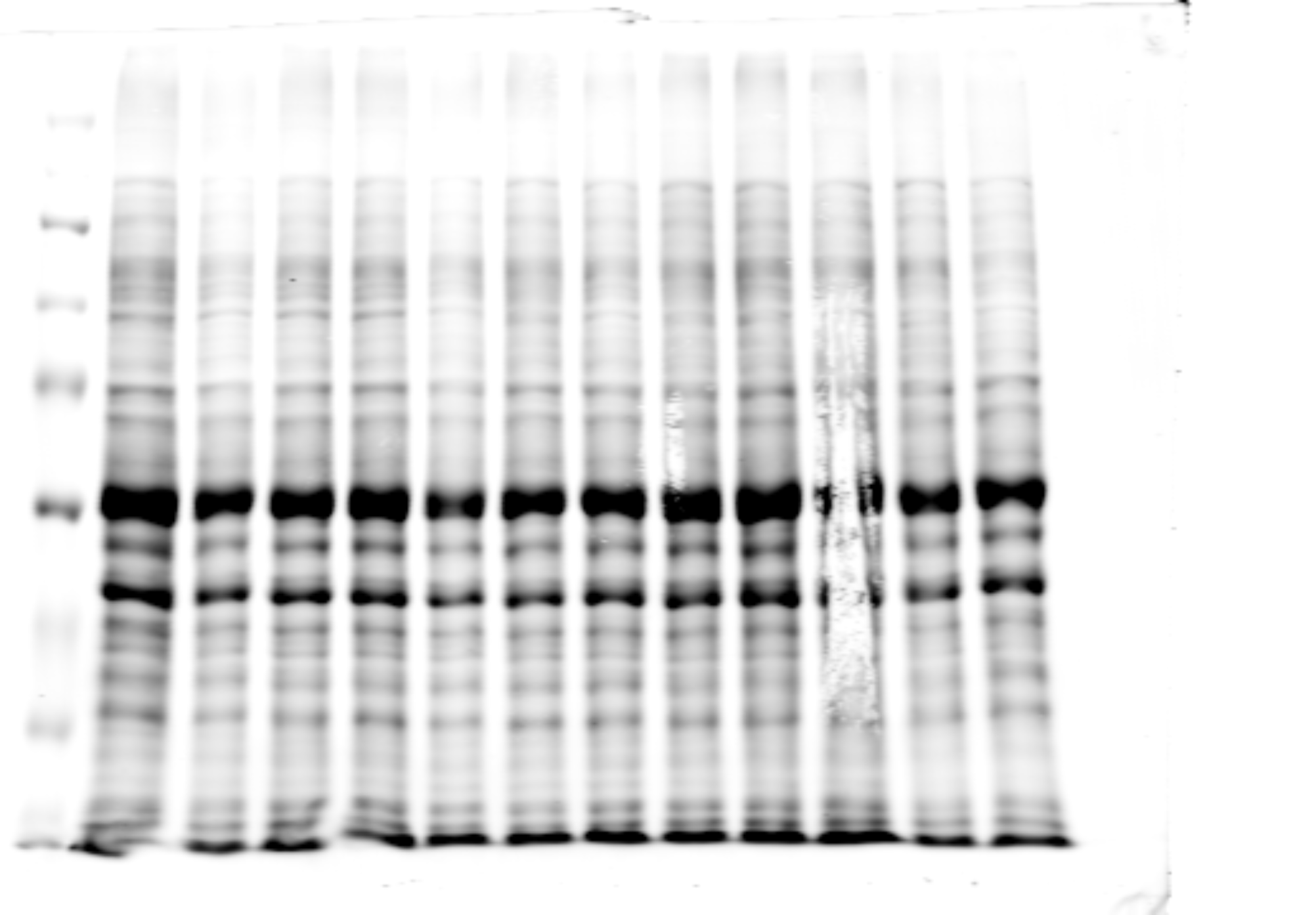

Supplement: Figure 2—figure supplement 1—source data 1. [file elife-78847-fig2-figsupp1-data1.zip › Figure2-supplement1-source data 1/Figure2_supplement1_source_data1_supplement1b_REVERT.tif]

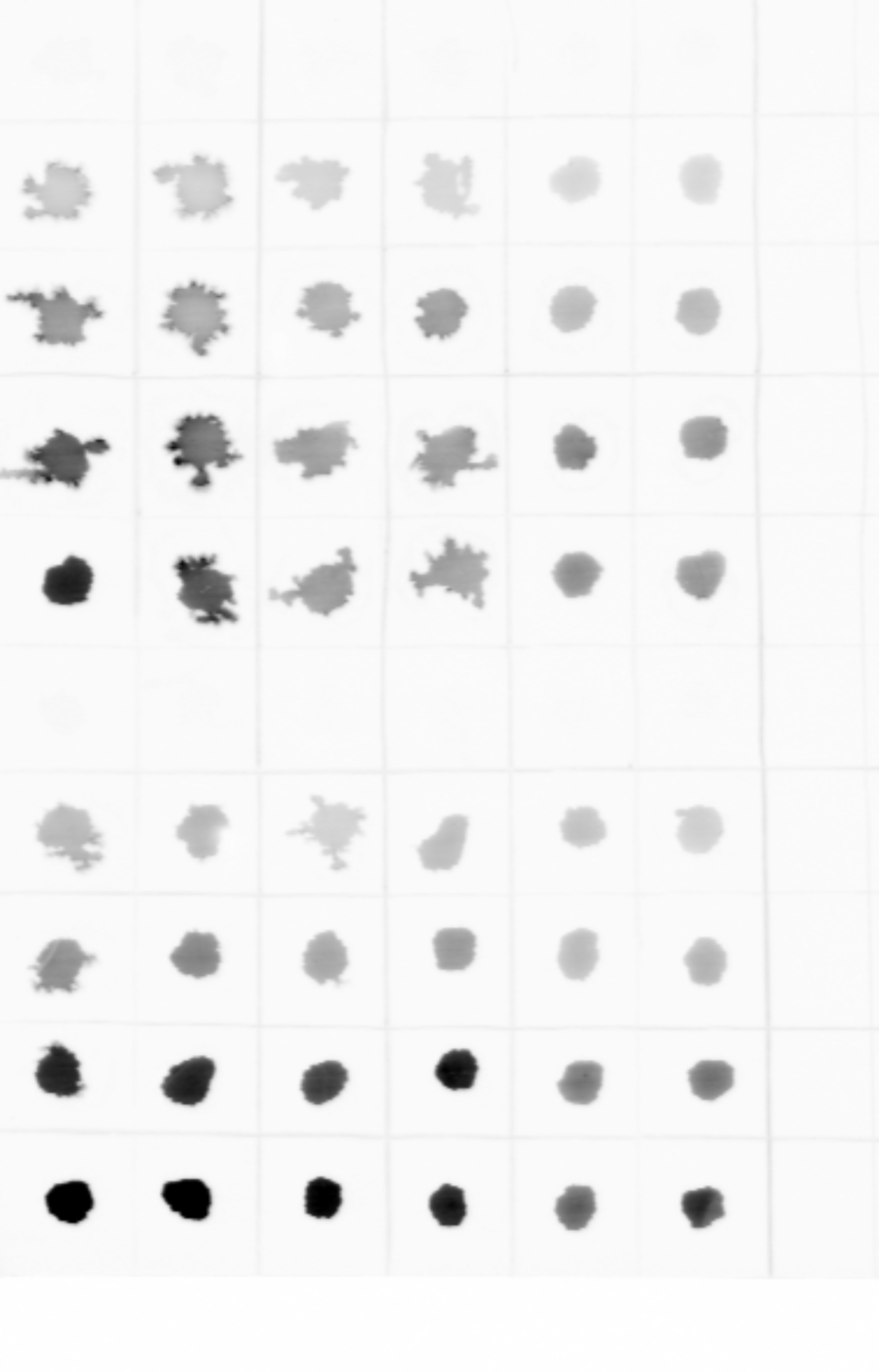

Supplement: Figure 2—figure supplement 1—source data 1. [file elife-78847-fig2-figsupp1-data1.zip › Figure2-supplement1-source data 1/Figure2_supplement1_source_data1_supplement1a_streptavidin.tif]

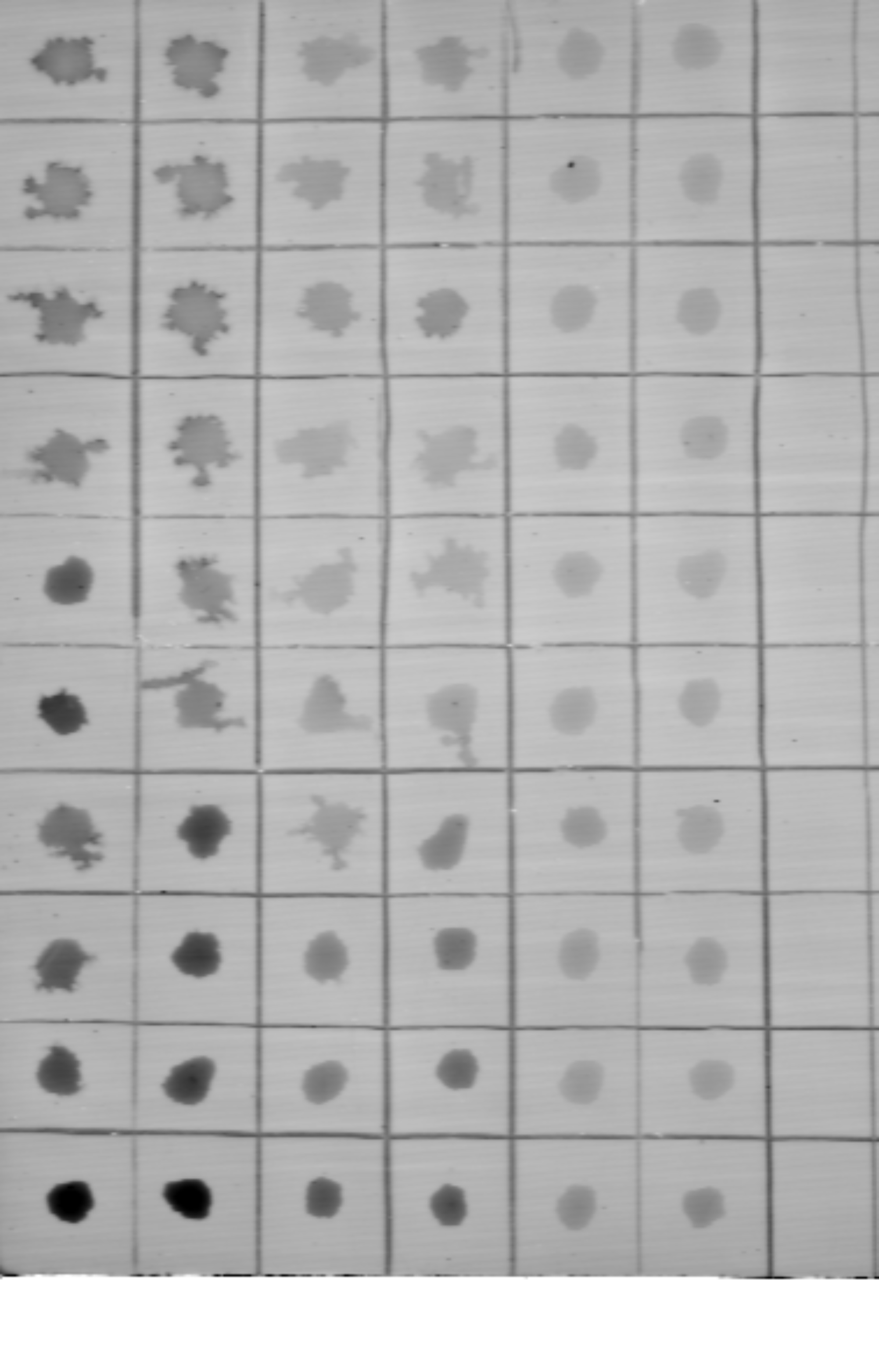

Supplement: Figure 2—figure supplement 1—source data 1. [file elife-78847-fig2-figsupp1-data1.zip › Figure2-supplement1-source data 1/Figure2_supplement1_source_data1_supplement1a_REVERT.tif]

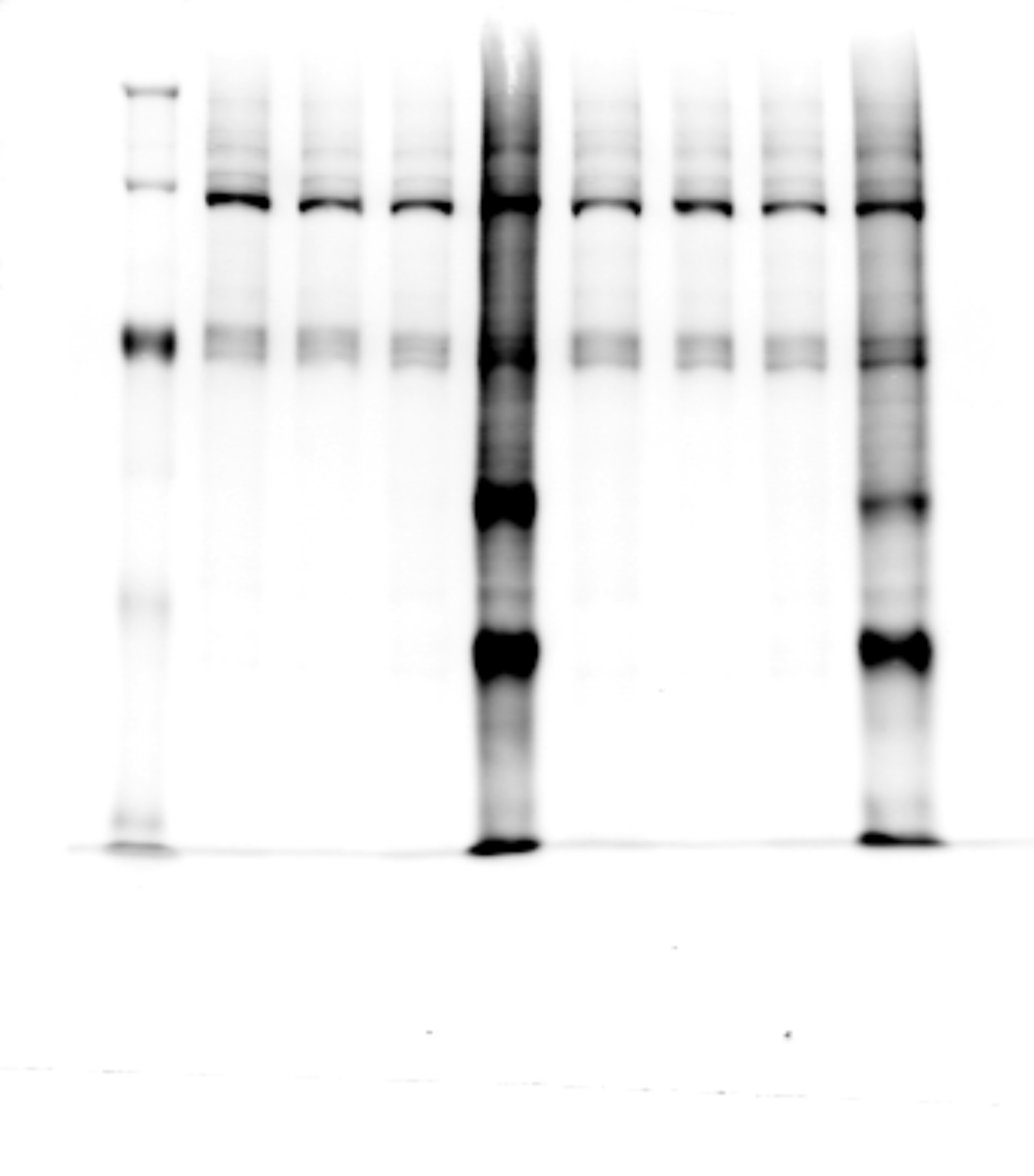

Supplement: Figure 3—figure supplement 1—source data 1. [file elife-78847-fig3-figsupp1-data1.zip › Figure3-supplement1-source data 1/Figure3_supplement1_source_data1_supplement1a_streptavidin.tif]

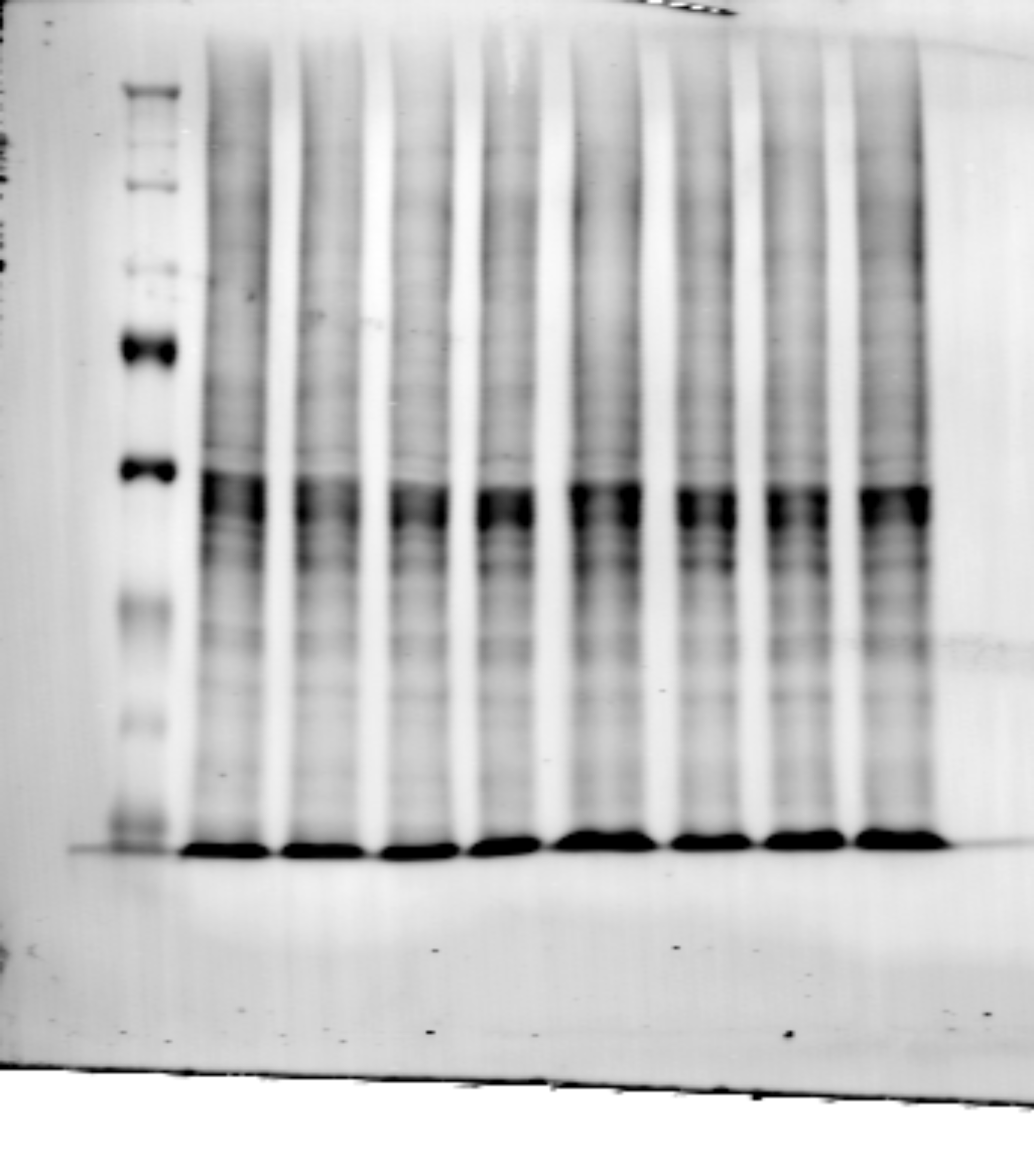

Supplement: Figure 3—figure supplement 1—source data 1. [file elife-78847-fig3-figsupp1-data1.zip › Figure3-supplement1-source data 1/Figure3_supplement1_source_data1_supplement1a_REVERT.tif]

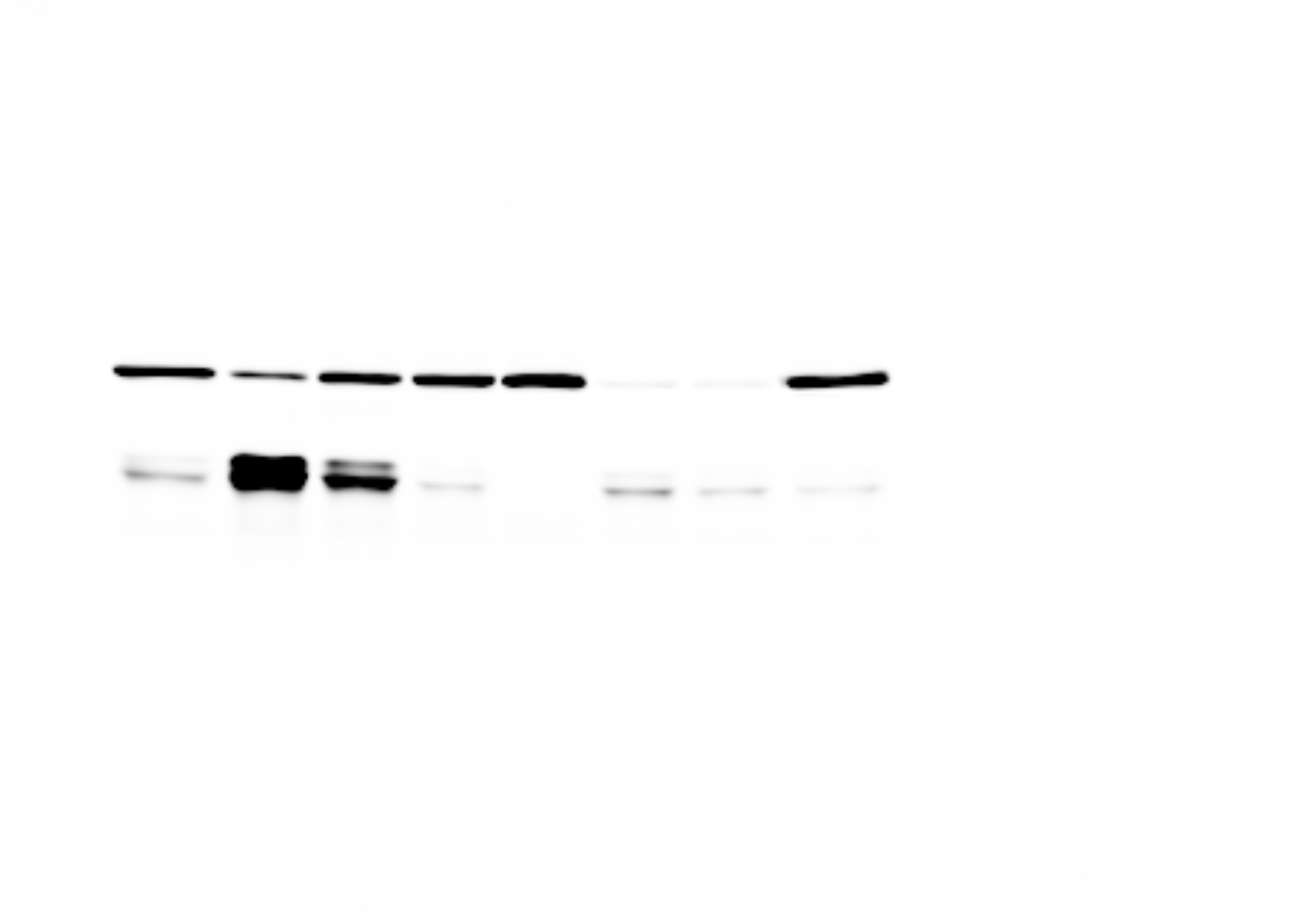

Supplement: Figure 3—figure supplement 1—source data 1. [file elife-78847-fig3-figsupp1-data1.zip › Figure3-supplement1-source data 1/Figure3_supplement1_source_data1_supplement1e-f_top_Th_bottom_dcx.tif]

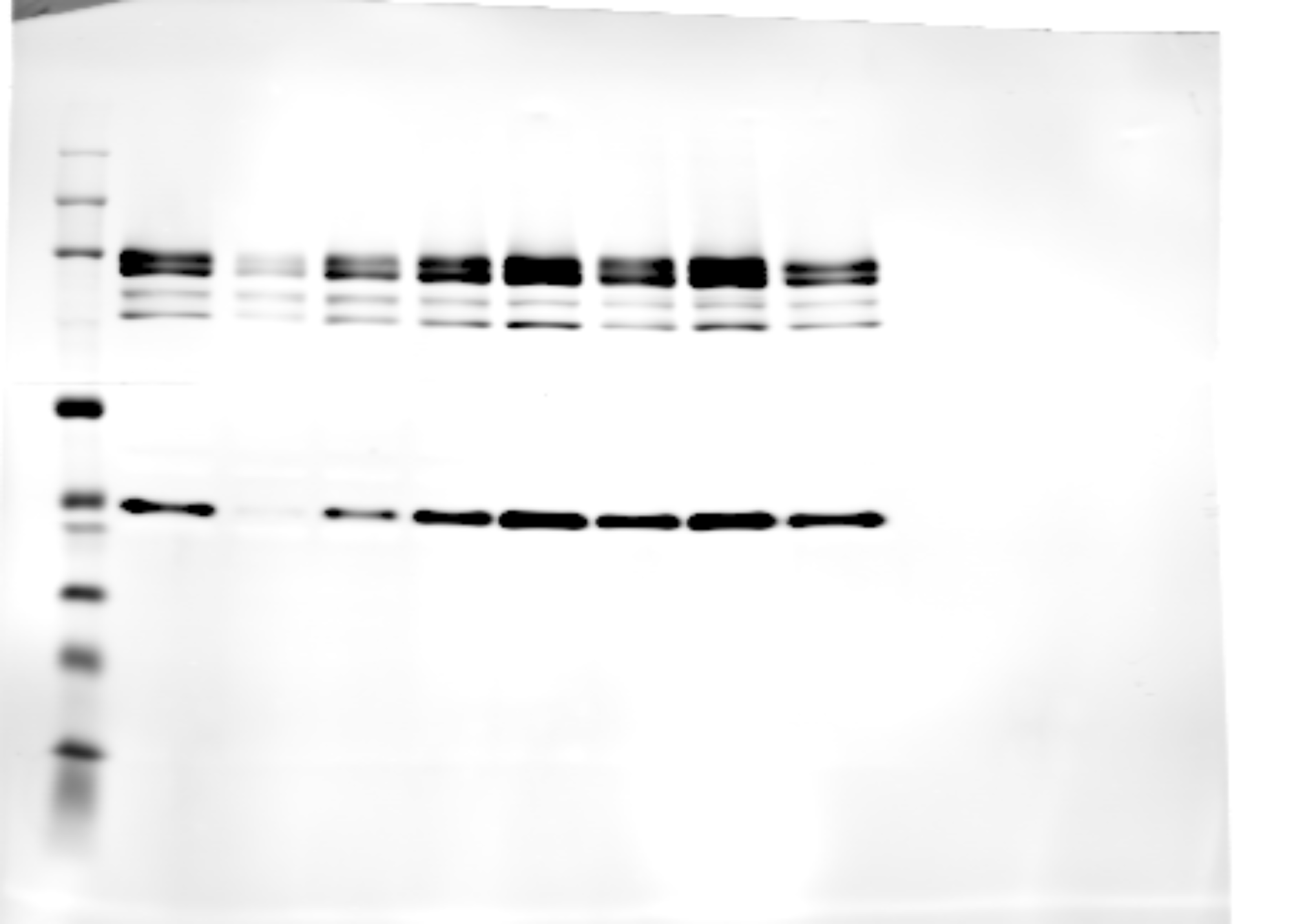

Supplement: Figure 3—figure supplement 1—source data 1. [file elife-78847-fig3-figsupp1-data1.zip › Figure3-supplement1-source data 1/Figure3_supplement1_source_data1_supplement1e-f_top_Psd95_bottom_synaptophysinI.tif]

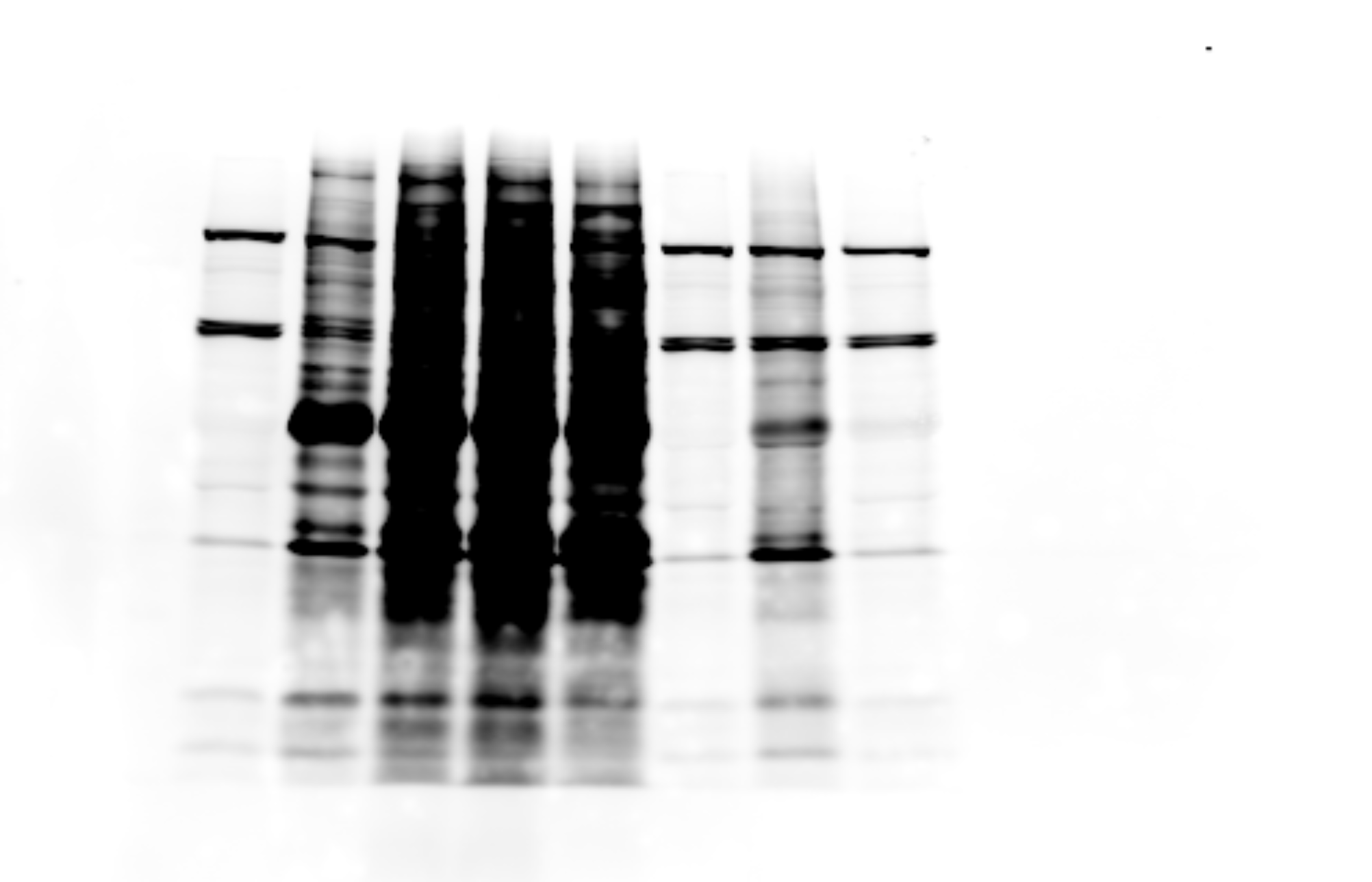

Supplement: Figure 3—figure supplement 1—source data 1. [file elife-78847-fig3-figsupp1-data1.zip › Figure3-supplement1-source data 1/Figure3_supplement1_source_data1_supplement1e-f_streptavidin_overexposed.tif]

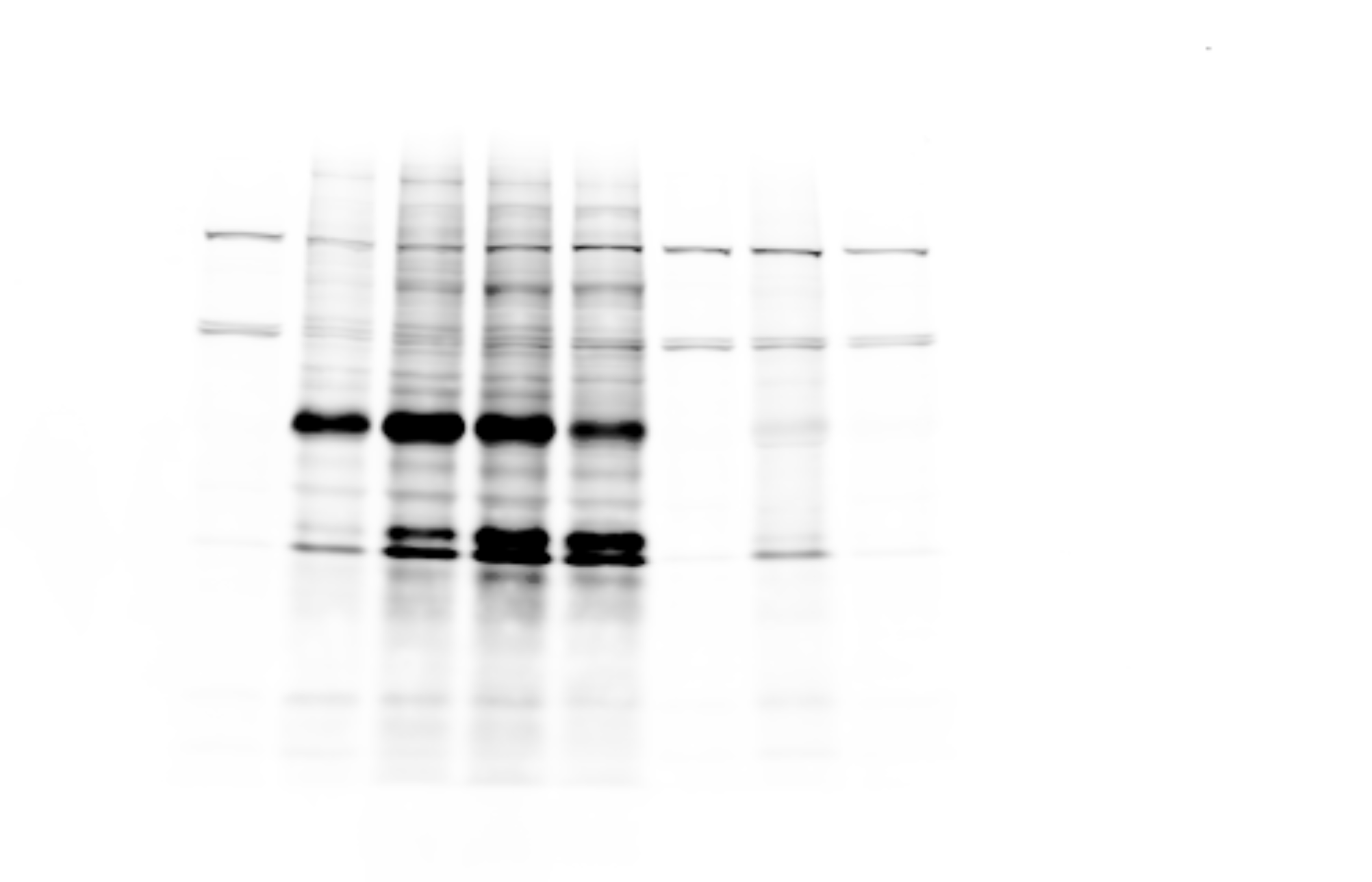

Supplement: Figure 3—figure supplement 1—source data 1. [file elife-78847-fig3-figsupp1-data1.zip › Figure3-supplement1-source data 1/Figure3_supplement1_source_data1_supplement1e-f_streptavidin.tif]

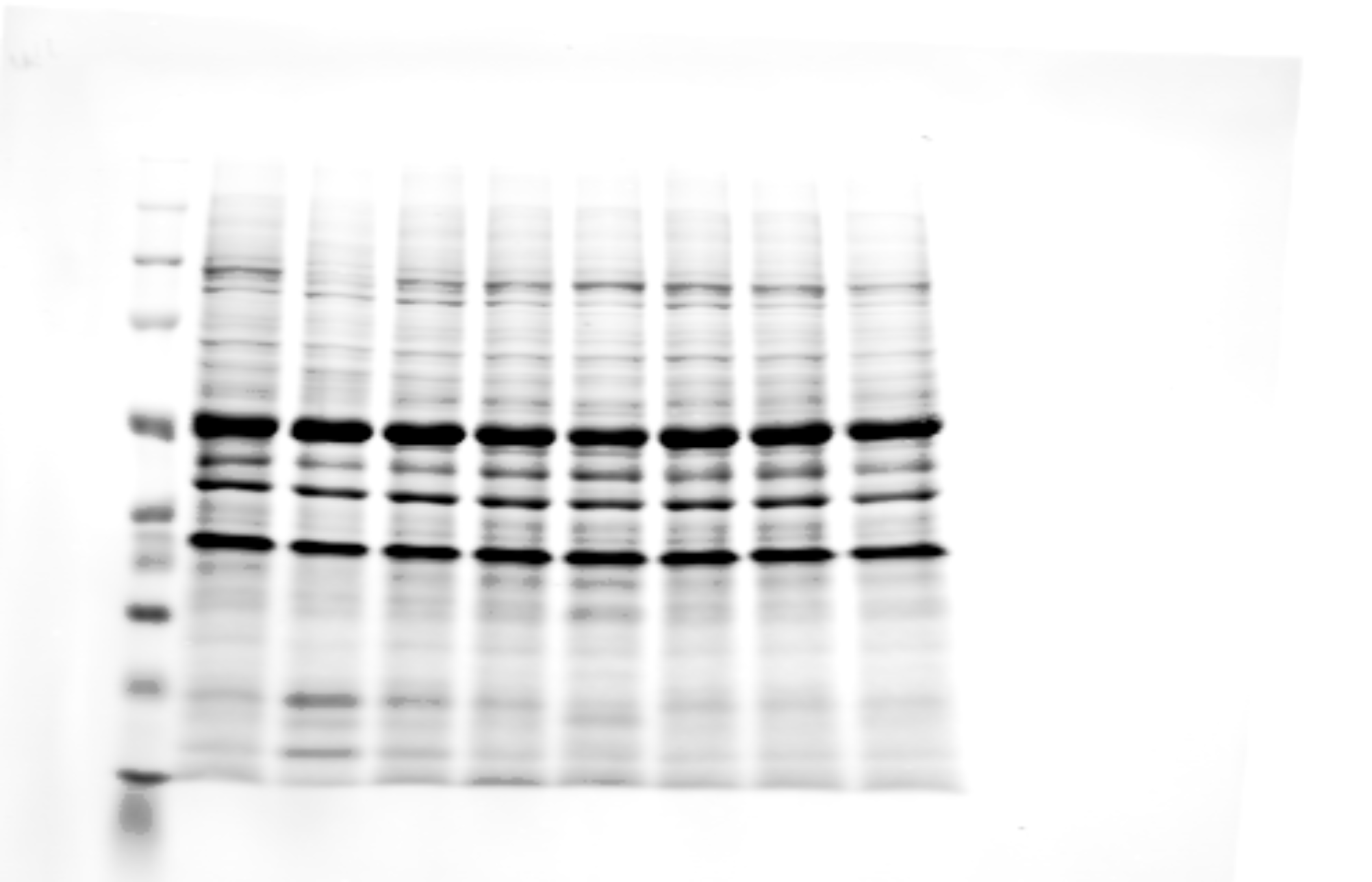

Supplement: Figure 3—figure supplement 1—source data 1. [file elife-78847-fig3-figsupp1-data1.zip › Figure3-supplement1-source data 1/Figure3_supplement1_source_data1_supplement1e-f_REVERT.tif]

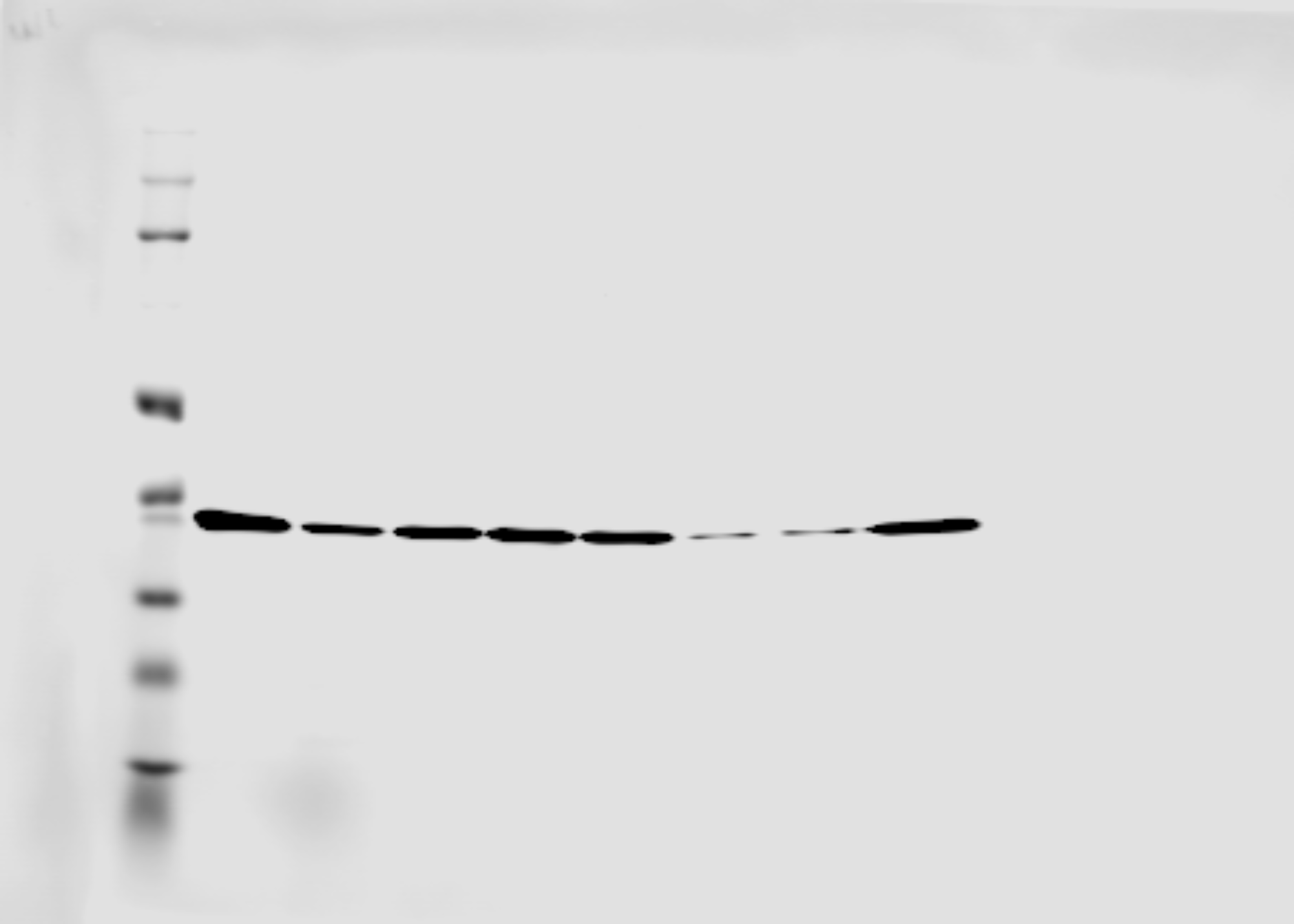

Supplement: Figure 3—figure supplement 1—source data 1. [file elife-78847-fig3-figsupp1-data1.zip › Figure3-supplement1-source data 1/Figure3_supplement1_source_data1_supplement1e-f_Darpp32.tif]

Figure 3 - figure supplement 1a

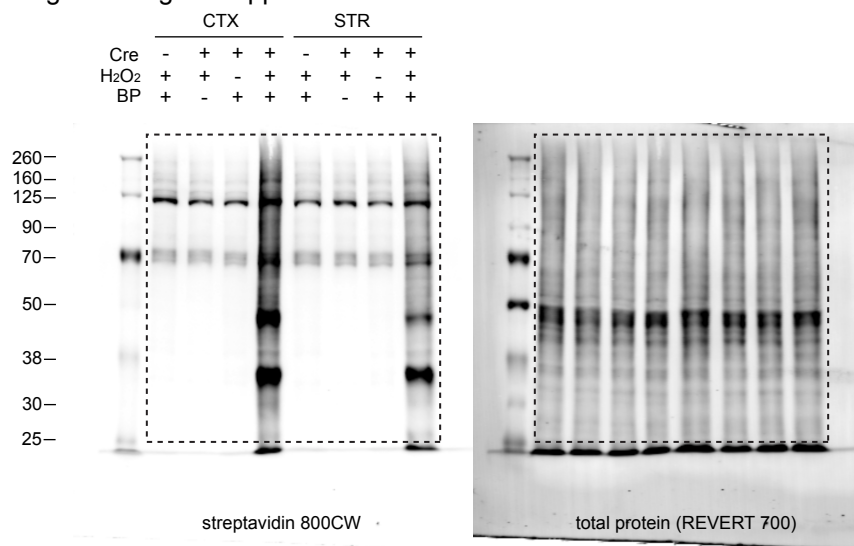

Figure 3b, and Figure 3 - figure supplement 1e

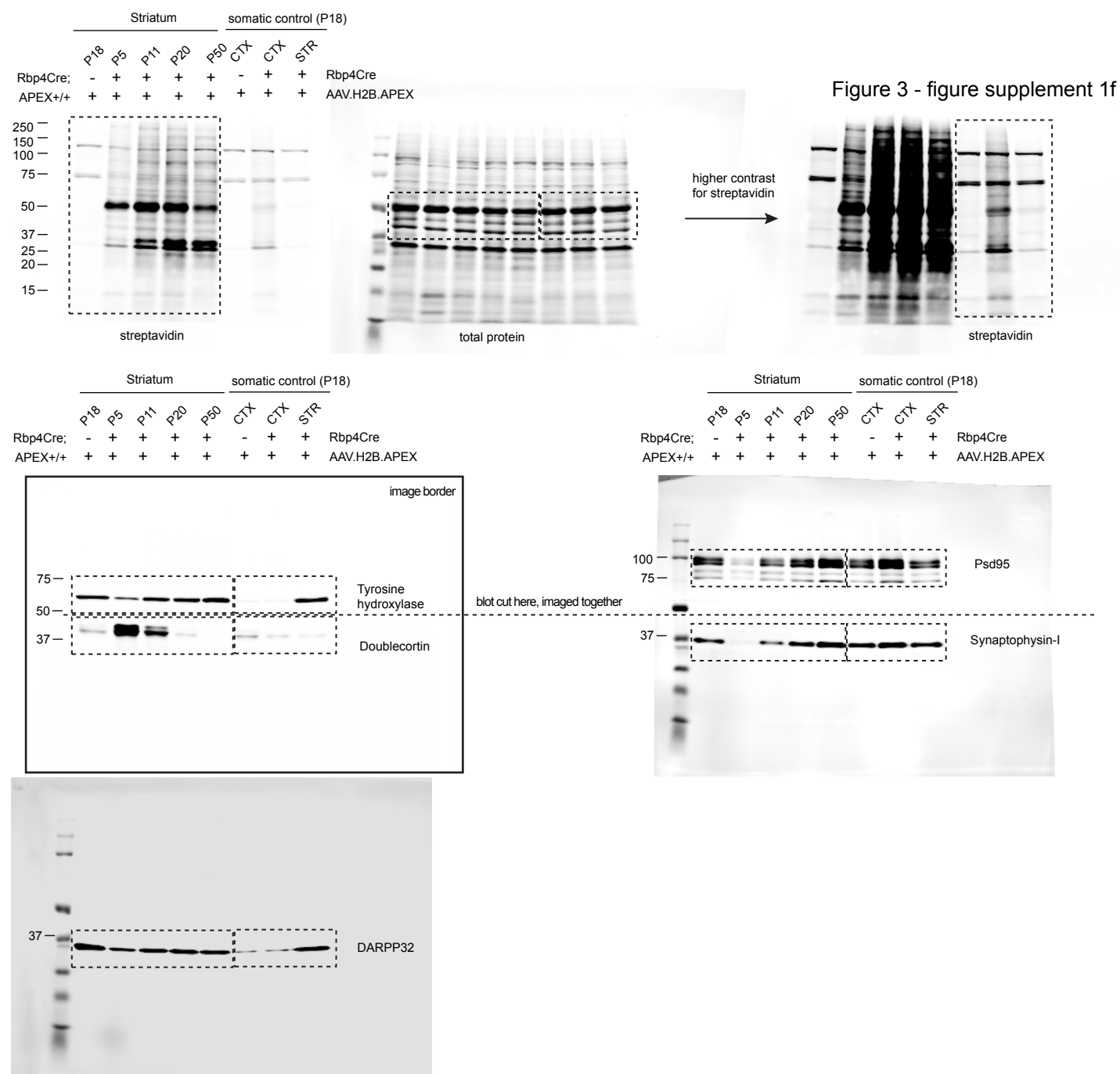

Supplement: Figure 3—figure supplement 1—source data 1. [file elife-78847-fig3-figsupp1-data1.zip › Figure3-supplement1-source data 1/Figure3_supplement1_source_data1.pdf]
