# Supplementary material for: Dynamic proteomic and phosphoproteomic atlas of corticostriatal axons in neurodevelopment
Source: eLife. 2022 Oct 14;11:e78847. doi: 10.7554/eLife.78847 (PMC9629834; doi:10.7554/eLife.78847)

Figure 2 - figure supplement 1a

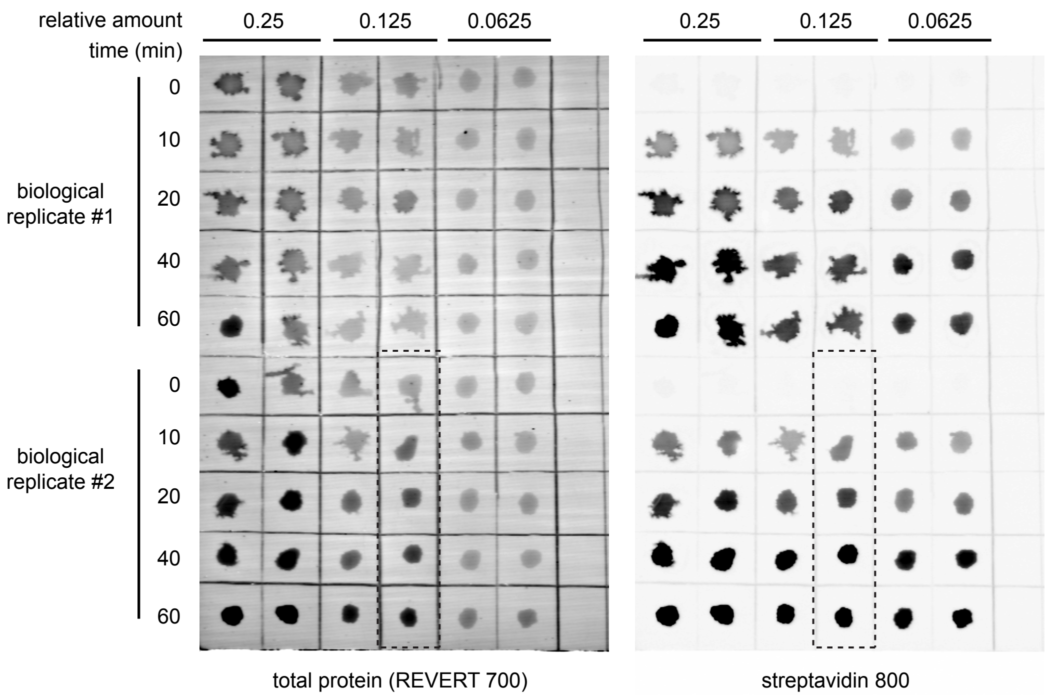

each sample was dotted twice (technical replicate for averaging) next to each other

Figure 2 - figure supplement 1b

Replicate 1

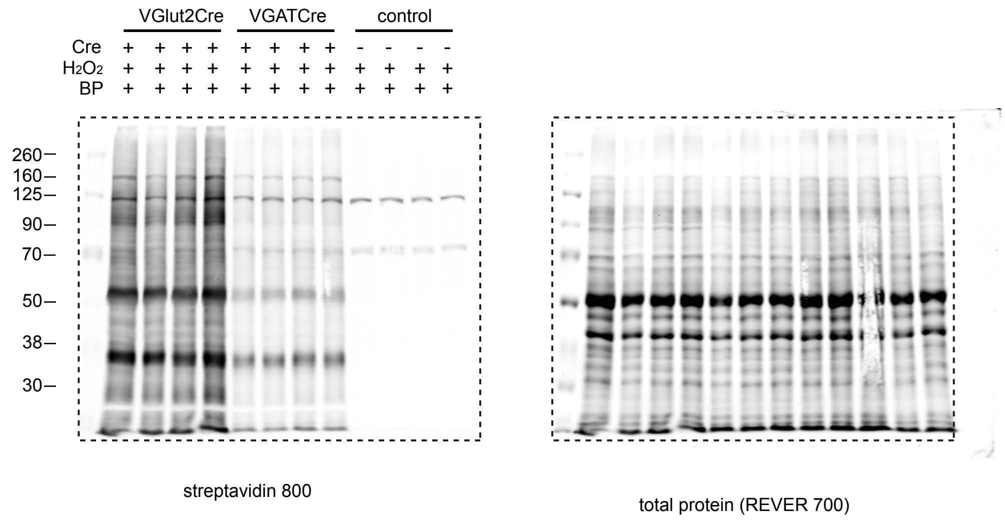

Supplement: Figure 2—figure supplement 1—source data 1. [file elife-78847-fig2-figsupp1-data1.zip › Figure2-supplement1-source data 1/Figure2_supplement1_source_data1.pdf]
